# Long non-coding RNA repertoire and open chromatin regions constitute midbrain dopaminergic neuron - specific molecular signatures

**DOI:** 10.1038/s41598-018-37872-1

**Published:** 2019-02-05

**Authors:** J. Gendron, C. Colace-Sauty, N. Beaume, H. Cartonnet, J. Guegan, D. Ulveling, C. Pardanaud-Glavieux, I. Moszer, H. Cheval, P. Ravassard

**Affiliations:** Inserm U 1127, CNRS UMR 7225, Sorbonne Universités, UPMC Univ Paris 06 UMR S 1127, Institut du Cerveau et de la Moelle épinière, ICM, F-75013 Paris, France

## Abstract

Midbrain dopaminergic (DA) neurons are involved in diverse neurological functions, including control of movements, emotions or reward. In turn, their dysfunctions cause severe clinical manifestations in humans, such as the appearance of motor and cognitive symptoms in Parkinson’s Disease. The physiology and pathophysiology of these neurons are widely studied, mostly with respect to molecular mechanisms implicating protein-coding genes. In contrast, the contribution of non-coding elements of the genome to DA neuron function is poorly investigated. In this study, we isolated DA neurons from E14.5 ventral mesencephalons in mice, and used RNA-seq and ATAC-seq to establish and describe repertoires of long non-coding RNAs (lncRNAs) and putative DNA regulatory regions specific to this neuronal population. We identified 1,294 lncRNAs constituting the repertoire of DA neurons, among which 939 were novel. Most of them were not found in hindbrain serotonergic (5-HT) neurons, indicating a high degree of cell-specificity. This feature was also observed regarding open chromatin regions, as 39% of the ATAC-seq peaks from the DA repertoire were not detected in the 5-HT neurons. Our work provides for the first time DA-specific catalogues of non-coding elements of the genome that will undoubtedly participate in deepening our knowledge regarding DA neuronal development and dysfunctions.

## Introduction

Midbrain dopaminergic (DA) neurons account for the majority of DA neurons in the adult brain^1,2^. They are mostly located within two structures, the *substantia nigra pars compacta* (SNpc) and the ventral tegmental area (VTA), both originating from the ventral mesencephalon during development. DA neurons from the SNpc project to the dorsolateral striatum and caudate putamen, thereby forming the nigrostriatal pathway involved in the control of voluntary movements. Their progressive but massive neurodegeneration in Parkinson’s Disease is responsible for the appearance of the motor symptoms that principally include rigidity, bradykinesia and tremor^3,4^. DA neurons from the VTA participate to the mesocorticolimbic pathway, associated with emotion and reward, as they innervate the ventral striatum and prefrontal cortex. Dysfunctions of these neurons have been linked to several human pathologies, in particular schizophrenia, depression and drug addiction^5^. Moreover, DA neurons from the VTA also degenerate in Parkinson’s Disease but to a lesser extent than the SNpc neurons^6–8^.

An increasing number of studies highlight the diversity of the midbrain DA neuronal subtypes from the molecular to the electrophysiological levels, not only between SNpc and VTA, but also within these two structures^9–12^. Thus, using single cell RNA-seq to identify cell-specific molecular signatures, it has recently been shown that adult midbrain DA neurons are subdivided into five subtypes that arise from only two populations of embryonic DA neurons^9^. Importantly, these two fetal subtypes do not each give rise to the SNpc or the VTA, but both participate to the emergence of these adult midbrain regions. In terms of spatial organization however, embryonic DA neurons segregate into two populations that will each define the future SNpc and VTA^13^. This spatial distribution occurs after the radial migration of differentiating DA neurons from the ventricular zone to the mantle layer of the ventral mesencephalon, creating an intermingled pool of DA neurons that will later constitute the SNpc and the VTA. Then, from gestation day 14.5 to 15.5 (E14.5–E15.5) SNpc neurons migrate tangentially, creating a spatial subdivision between the midbrain DA neurons in mouse embryos^14^. Therefore at stage E14.5, mesencephalic DA neurons constitute a roughly spatially and molecularly homogeneous population, suggesting that this embryonic stage constitutes a developmental crossroad before the important diversification of DA neuronal subsets.

So far, molecular signatures defining DA neuronal subtypes have been obtained using transcriptomic data only focused on protein-coding genes^9,10^. However, recent developments suggest that non-coding elements of the genome such as long non-coding RNAs (lncRNAs) or active regulatory sequences, including promoters or enhancers, constitute repertoires displaying a greater cell specificity than protein-coding genes^15–20^. LncRNAs are increasingly scrutinized for their multiple regulatory functions from the epigenetic to the post-translational levels^21–24^, and for their involvement in crucial developmental and cellular processes, such as neuronal differentiation^17,25–28^. Importantly, genetic mapping of single nucleotide polymorphisms (SNPs) in human pathologies demonstrated that the majority of the SNPs fall into non-coding regions^29,30^. Consistent with this observation, literature linking lncRNAs as well as active regulatory sequences to human diseases, including Alzheimer disease, Parkinson’s Disease, Schizophrenia, drug addiction, cancer, or Diabetes, is growing^17,31–40^.

In this study, we seek to expand our knowledge on the molecular signatures displayed by mesencephalic DA neurons at E14.5 before their divergence into specific cellular subtypes involved in many physiological and pathological mechanisms. We used high throughput RNA-seq and ATAC-seq and identified novel lncRNAs and active regulatory sequences specific from this population.

## Results

### Efficient enrichment in DA neurons from mouse E14.5 ventral mesencephalon

**Figure 1:**
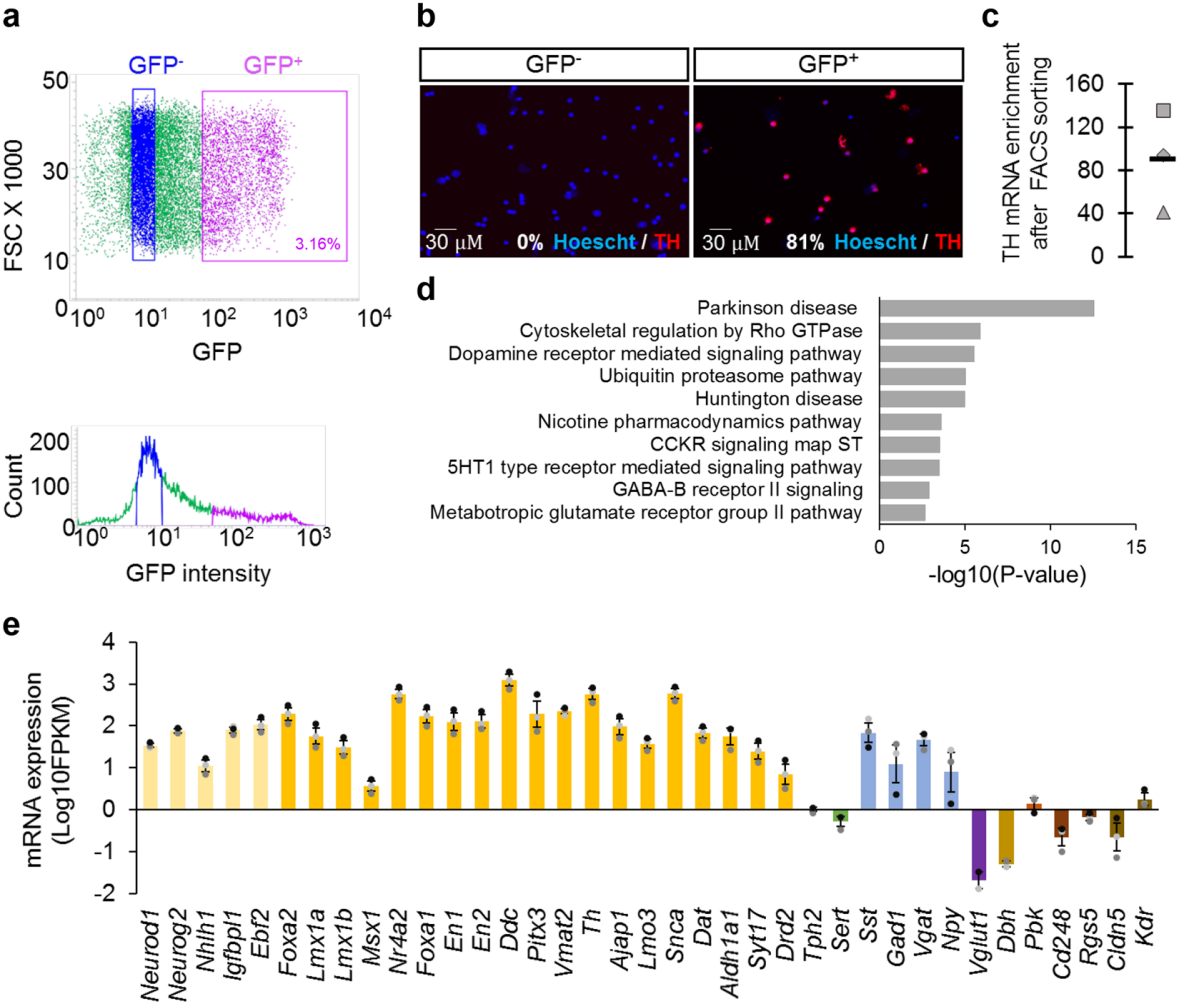
Protein-coding transcriptome of DA neurons isolated from ventral mesencephalons of E14,5 embryos expressing GFP in *Th*^+^ cells. (**a**) FACS sorting of GFP^+^ and GFP^−^ cell populations for subsequent RNA-seq and ATAC-seq analyses. (**b**) Th expression (red) assessed by immunofluorescence on GFP^+^ and GFP^−^ cells cultured for 90 minutes after FACS. Nuclei were stained using Hoechst (blue). (**c**) *Th* mRNA relative expression of 3 independent GFP^+^ cell populations used for RNA-seq (triangle, diamond and square), compared to their matching control GFP^−^ cell populations. The bar represents the mean of the 3 enrichment values. mRNA expression was normalized relatively to *Tbp* mRNA expression. (**d**) Pathway analysis (Panther 2016) performed on the 1500 most expressed protein-coding genes obtained from 3 independent RNA-seq datasets, excluding mitochondrial genes. (**e**) mRNA expression in Log10(FPKM) of numerous cellular subtype marker genes. Each circle represents mRNA expression of a marker gene from 1 RNA-seq, and the bar represents the mean of the 3 values. Error bars show standard error of the mean. The color code is as follow: pale yellow, dopaminergic progenitors; yellow, differentiating and differentiated dopaminergic neurons; green, serotonergic neurons; blue, GABAergic neurons; purple, glutamatergic neurons; mustard, noradrenergic neurons; brown, radial glial like cells; dark brown, pericytes; brown-orange, endothelial cells.

To ensure the cell-specificity of our DA neuronal population, we FACS-purified cells originating from E14.5 ventral mesencephalon of transgenic mice expressing GFP under the control of the rat Tyrosine Hydroxylase (*Th*) promoter^41^ (Fig. 1a). Cells from the sorted populations were either cultured for 90 minutes and assessed for Th expression, or used to carry out deep RNA-seq and ATAC-seq. Immunofluorescence experiments revealed 81% of Th^+^ cells in the GFP^+^ population, and no Th^+^ cells in the GFP^−^ population (Fig. 1b), consistent with previous data from the literature^41^. In parallel, *Th* mRNA expression was analysed by RT-qPCR prior to RNA-seq, showing a 90 fold enrichment in GFP^+^ cells compared to the GFP^−^ control population (Fig. 1c). We generated cDNA libraries from polyadenylated RNA and mapped ∼800 million paired-end sequence reads from a total of 3 independent RNA-seq datasets originating from the GFP^+^ cells (288,046,125 reads for the first dataset; 233,351,531 for the second dataset and 394,620,452 for the third). We performed a pathway analysis on the 1500 most expressed protein-coding transcripts obtained (Fig. 1d), excluding mitochondrial genes, and observed a strong enrichment in genes associated with the terms “Parkinson’s Disease” (p-value = 2.874 × 10^−13^) and “Dopamine receptor-mediated signalling pathway” (p-value = 2.862 × 10^−6^), that appeared within the first 3 occurrences. Terms associated with other neurotransmitter systems, such as serotonergic (5-HT), GABAergic and glutamatergic receptors signalling pathways, also emerged from this analysis pathway, yet with a much less significant p-value. Accordingly, based on fragments per kilobase per million of reads (FPKM), DA lineage marker genes, from progenitors to differentiated cells, were strongly expressed (Fig. 1e), in contrast with marker genes from glutamatergic, noradrenergic, 5-HT neurons and from pericytes, radial glial like cells and endothelial cells that constitute non-neuronal cell types present in the E14.5 ventral mesencephalon^9^. GABAergic markers were however highly expressed, suggesting a slight contamination of our DA population by GABAergic neurons. This GABAergic population potentially covers the 20% Th^−^ cells observed in the FACS-sorted GFP^+^ population (Fig. 1b). Overall, the transcriptomic data, along with Th expression analyses at the protein and mRNA levels, indicate that we predominantly isolated DA neurons from E14.5 ventral mesencephalon. RNA-seq and ATAC-seq performed using this approach therefore constitute relevant tools to identify the DA repertoires of lncRNAs and active regulatory sequences.

### The lncRNA repertoire of mesencephalic DA neurons

**Figure 2:**
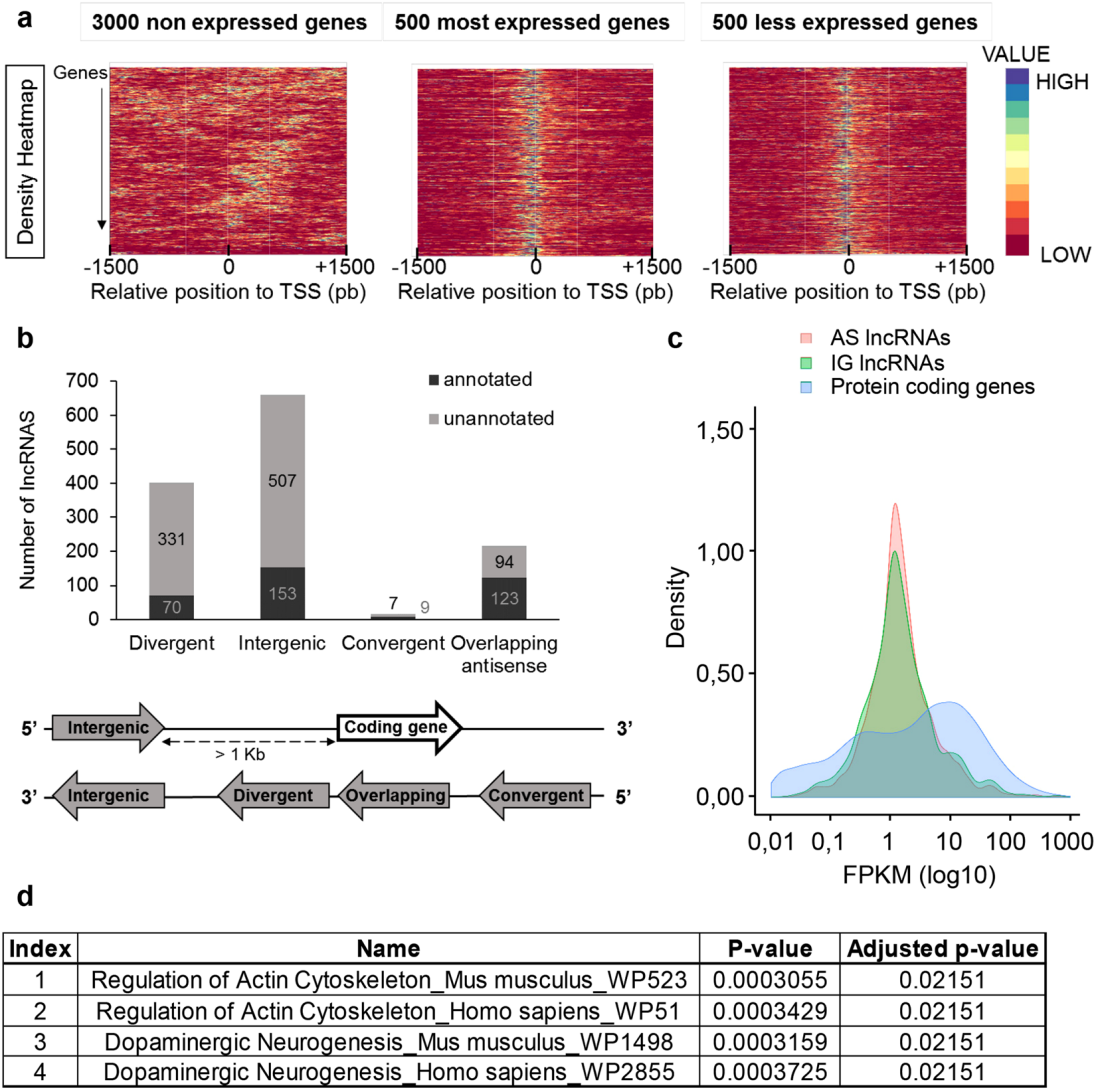
Identification of the lncRNAs expressed in mesencephalic DA neurons. (**a**) Density heatmaps representing the height of ATAC-seq peaks relative to the TSS position of non-expressed (left), highly (middle) or weakly (right) expressed genes in FACS-purified DA neurons. (**b**) Number of lncRNAs depending on their categories as represented below the graph. Dark grey, lncRNAs annotated in Ensembl; light grey, lncRNAs unannotated. (**c**) Density plot of the coding genes described in Ensembl and the lncRNAs (AS: antisense, IG: intergenic). (**d**) Pathway analysis (Wikipathway 2016, Enrichr) performed on the neighbouring genes to the top 20% most expressed lncRNAs using 3 independent RNA-seq datasets.

Identification of lncRNAs relied on the following criteria: i) length ≥ 200 pb, ii) expression ≥ 1 FPKM for at least one out of the 3 RNA-seq datasets and at least 2 non-null FPKM values out of the 3 RNA-seq replicates, as well as iii) low protein-coding potential as assessed by CPAT^42^. From the list of lncRNAs obtained using these parameters, we identified two categories of transcripts depending on the presence or the absence of an ATAC-seq peak at the transcription start site (TSS). Indeed, we observed that the presence of ATAC-seq peaks at TSS correlates with genes actively transcribed, independent of the level of expression (Fig. 2a). Nevertheless, sequencing polyadenylated RNA often induces a bias towards less mapped reads in the first exons, especially for long transcripts as clearly illustrated in Supplementary Fig. S1. Therefore we also took in account transcripts with multiple exons that fulfilled the first 3 criteria, but that were not associated with open chromatin at their putative TSS, in order to keep lncRNAs whose first exon(s) had potentially not been correctly sequenced. In contrast with multiexonic transcripts with identified junctions between exons, the likelihood to confuse unannotated monoexonic transcripts with transcriptional background, or even sequencing artefacts led us to discard monoexonic transcripts not associated with an ATAC-seq peak at their TSS. Finally, in situations where several isoforms were identified, we only took into account the most expressed isoform. Using the combination of all of the above criteria, the list of selected transcripts was defined as repertoire of lncRNAs. This way we identified 1,294 lncRNAs, of which 939 had not yet been annotated (Fig. 2b). We used a previously described classification to categorize lncRNAs^17^, and found 660 “intergenic”, 401 “divergent”, 217 “overlapping antisense” and 16 “convergent” lncRNAs as described in the scheme Fig. 2b. Amongst the unannotated lncRNAs, 73.1% carried a single exon, whereas monoexonic lncRNAs constituted only 16.9% of the annotated lncRNAs pool (data not shown). Interestingly, in the first 100 most expressed lncRNAs from the mesencephalic DA repertoire, 37 were novel transcripts and 16 of them had only one exon (Supplementary Table S1). Consistent with the literature^16,17,22,24,43^, we found that lncRNAs were weakly expressed compared to protein-coding genes, with no overt difference between intergenic lncRNAs and the other lncRNAs categories (grouped as “Antisense” in the Fig. 2c). Using lncRNAs’ closest upstream and downstream genes, or in the case of the overlapping antisense lncRNAs, their overlapped genes, we performed a pathway analysis on the top 20% most expressed lncRNAs from this and found “Dopaminergic Neurogenesis” (adjusted p-value = 0.02151) amongst the only two significantly enriched terms (Fig. 2d). In addition to the fact that 72.6% of the transcripts identified were not previously annotated, this strongly suggests that this DA lncRNA repertoire reflects a high degree of specificity associated with DA neurons.

In order to examine this remarkable tissue specificity further, we compared the DA repertoire of lncRNAs to a repertoire generated at the same stage from hindbrain serotonergic (5-HT) neurons, a monoaminergic neuronal subtype close to DA neurons^44^. Mesencephalic DA neurons and hindbrain 5-HT neurons originate from each side of the mid-hindbrain organizer, and are distributed within anatomically very close nuclei. Both these monoaminergic neurons display a similar developmental pattern regarding kinetics of progenitor specification, migration or differentiation, and project in numerous common brain areas. Importantly, 5-HT neurons also degenerate in Parkinson’s Disease and have been associated with some motor symptoms such as resting tremors, but also non motor symptoms, including anxiety and depression^45^. Thus, using the same approach, we took advantage of Masch1 CRE X Rosa YFP mice that express YFP in 5-HT neurons^46^, to FACS-purify YFP cells from the rhombomeres 1 to 3 of E14.5 embryos (Supplementary Fig. S2a). Following FACS-sorting, tryptophane hydroxylase 2 (Tph2), the neuronal rate limiting enzyme of serotonin biosynthesis, was used as marker. Tph2 immunofluorescence demonstrated an enrichment of 5-HT neurons in the YFP^+^ population, containing 98% of Tph2^+^ cells, compared to the YFP^−^ population which exhibited 24% of Tph2^+^ cells (Supplementary Fig. S2b). *Tph2* mRNA expression, analysed by RT-qPCR prior to RNA-seq, indicated a 16.25 fold enrichment in YFP^+^ cells compared to the YFP^−^ control population (Supplementary Fig. S2c). Regarding the RNA-seq, cDNA libraries from polyadenylated RNA were produced and about 500 million paired-end sequence reads were mapped in the totality of the 3 independent datasets generated (239,852,552 reads for the first dataset; 199,165,019 for the second dataset and 155,506,770 for the third). Sequencing revealed a high expression of 5-HT marker genes in FPKM, but also of genes expressed in DA and GABAergic neurons (Supplementary Fig. S2d). However, principal component analysis comparing the RNA-seq datasets obtained with the FACS-sorted cells from E14.5 ventral mesencephalons and from rhombomeres r1-3, confirmed that they constitute 2 distinct cell populations, with the replicates from each cell type forming 2 separate clusters (Supplementary Fig. S3). Using all criteria described previously, we identified in the 5-HT cell population a repertoire of 1,293 lncRNAs, among which 806 had not yet been annotated (Supplementary Fig. S4). We specifically found 594 “intergenic”, 14 “divergent”, 551 “overlapping antisense” and 134 “convergent” lncRNAs. Since the libraries of 5-HT neuron RNA-seq experiments were not performed in a stranded-specific manner, we could not infer the strand for unannotated monoexonic transcripts. As our strategy was to discard transcripts lying within 1 kb from a protein-coding gene on the same strand (see material and methods), this led us to discard all unannotated monoexonic transcripts located at less than 1 kb from a protein-coding gene for the 5-HT repertoire. This probably explains the relative low number of divergent lncRNAs in 5-HT neurons compared to the DA repertoire (Fig. 2b).

The 168 remaining monexonic transcripts were mostly intergenic (located at a distance above 1 kb from a protein coding gene, n = 139) and unannotated (only 4 were already annotated). Moreover, we observed an elevated number of antisense transcripts in the 5-HT repertoire (n = 551) compared to the DA repertoire (n = 217), with the number of annotated transcripts in the 5-HT repertoire (n = 265) even higher than the total number of DA antisense transcripts (see Figs 2 and S4). Since the identification of such annotated lncRNAs is not impacted by the difference of library preparation (stranded *versus* non-stranded), this indicates that the elevated number of antisense transcripts in the 5-HT repertoire reflects a distinctive cellular feature rather than a technological bias.

**Figure 3:**
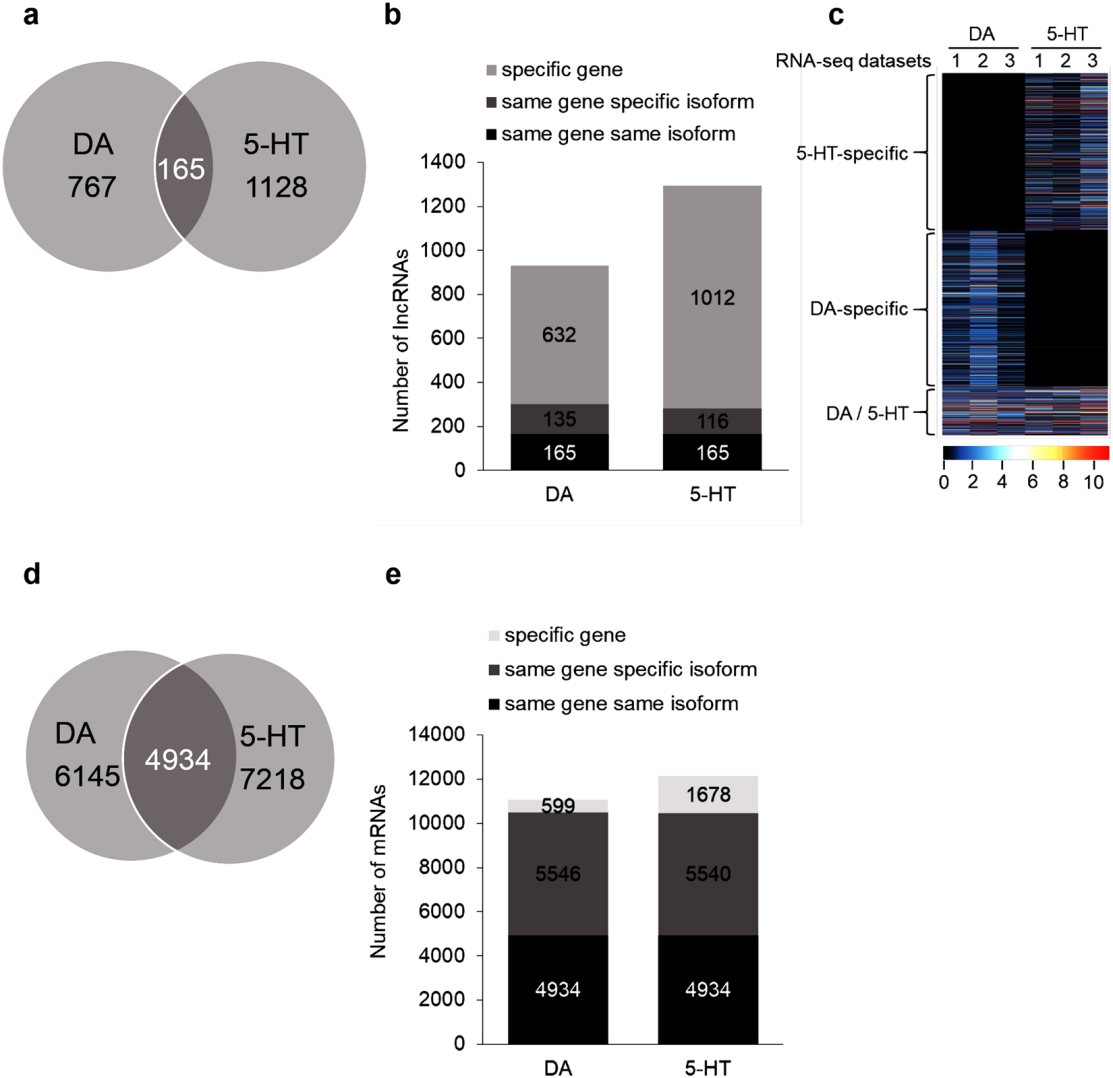
Comparison of the DA and 5-HT repertoires of lncRNAs and protein-coding mRNAs. (**a**) Venn diagram of overlap of both lncRNA repertoires. (**b**) Distribution of lncRNAs transcripts in the DA and 5-HT repertoires depending on their cell specificity. (**c**) Heatmap representing expression in FPKM of each transcripts from both repertoires in the DA and 5-HT datasets (3 samples each). (**d**) Venn diagram of overlap of both protein-coding mRNAs repertoire. (**e**) Distribution of protein-coding transcripts in the DA and 5-HT repertoires depending on their cell specificity.

To assess the specificity of DA and 5-HT lncRNA repertoires, we focused on categories of lncRNAs that were represented in both datasets. Thus, for unannotated monoexonic lncRNAs only intergenic transcripts were considered. We extracted 767 lncRNAs specific to DA neurons, 1128 specific to 5-HT neurons and 165 expressed in both cell types (Fig. 3a). Interestingly, common lncRNAs displayed higher expression level than cell-specific transcripts (Fig. 3c). In our methodology to establish the lncRNA repertoire of a given cell type, we only took in account the most expressed transcript when several isoforms of a same lncRNA were present. Therefore, we then evaluated the possibility that some specific lncRNAs may in fact have an equivalent, which would be a different isoform, in the other cell type. Thus, to compare isoform usage between DA and 5-HT samples, we looked at correspondence between lncRNAs expressed in the repertoires of both cell types. We thus defined three possible levels of specificity: “specific gene” when the lncRNA is present in one cell type only, “same gene specific isoform” in such case this isoform is seen only in one cell type but the other cell type expresses another isoform for the same gene, and “same gene same isoform” when the same isoform of the lncRNA is used in both cell types (namely, the 165 lncRNAs). Figure 3b shows the repartition of all three categories in DA and 5-HT neurons. In both cases, “specific gene” is the most important category by far. The same analysis on protein-coding transcripts demonstrated that the proportion of transcripts specifically expressed in one or another cell type is much lower than for the lncRNAs: 55.5% of specific mRNAs in DA neurons and 59.4% in 5-HT neurons *versus* 82.3 and 87% of specific lncRNAs in DA and 5-HT neurons respectively (Fig. 3a,d). Importantly, focusing on the gene level rather than the transcript level, only 5.4% protein-coding transcripts expressed in DA neurons and 13.8% in 5-HT neurons are cell type specific genes, whereas this proportion is much higher for lncRNAs (68 and 78%; Fig. 3b,e). Moreover, we noticed once again the predominance of novel lncRNAs in the DA- and 5-HT-specific repertoires (78% and 73% respectively), whereas the transcripts expressed in both neuronal subtypes were mostly previously annotated (90%). Altogether, these data highlight the high cell-specificity of the lncRNAs.

### Mapping open chromatin regions in mesencephalic DA neurons

**Figure 4:**
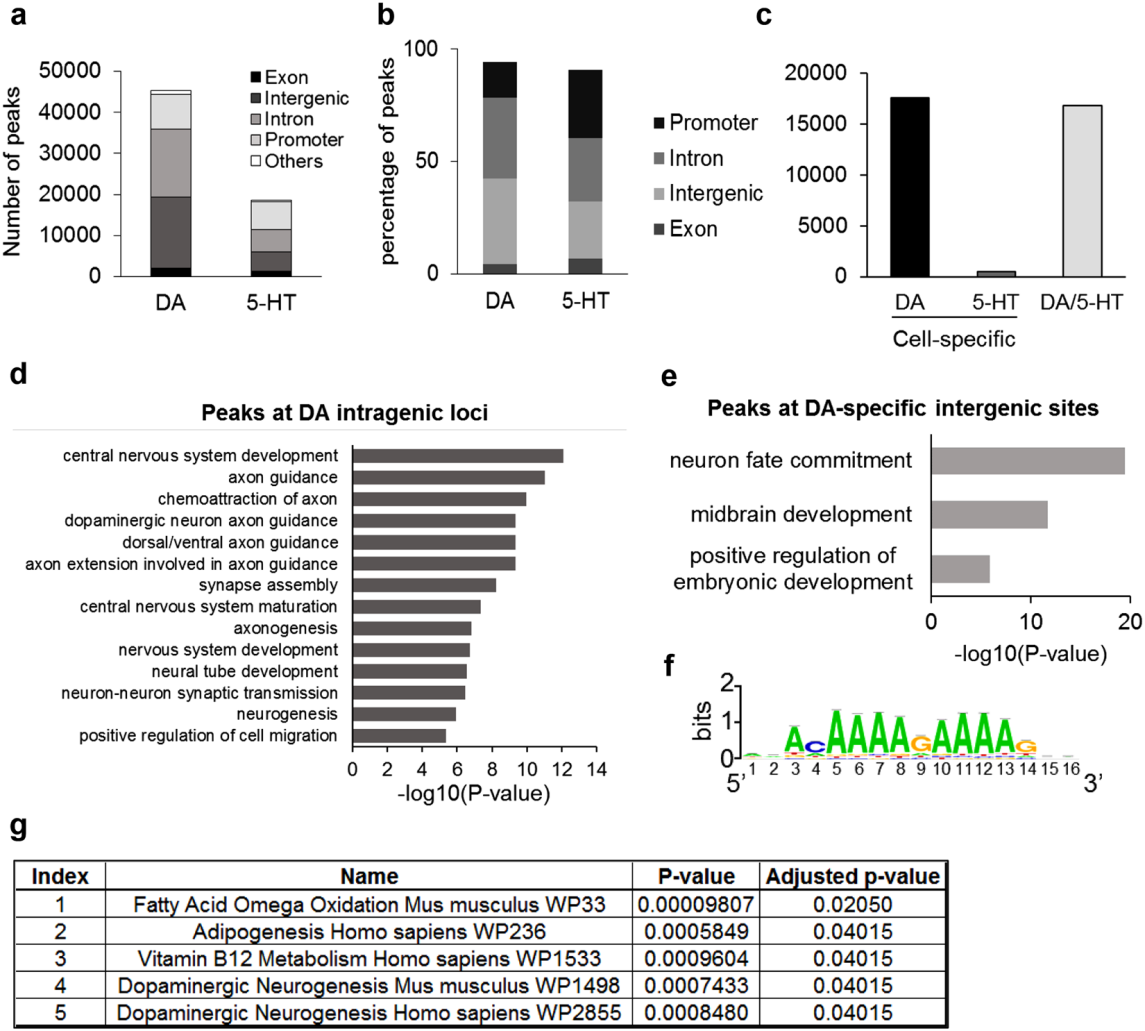
Analysis of DNA regulatory regions using ATAC-seq. (**a**) Number of ATAC-seq peaks depending on their genomic loci. n = 3 ATAC-seq datasets for the DA repertoire; n = 2 ATAC-seq datasets for the 5-HT repertoire. (**b**) Percentage of ATAC-seq peaks depending on their genomic loci. The category named ‘Others’ regroups ATAC-seq peaks found at 3′UTR regions and TTS. (**c**) Number of ATAC-seq peaks specific to the 3 DA datasets (DA), specific to the 2 5-HT datasets (5-HT), and present in the 5 datasets (DA/5-HT). (**d**) GO Biological process on ATAC-seq peaks linked to exons and introns from the DA-specific ATAC-seq repertoire (Enrichr). (**e)** GO Biological process on intergenic ATAC-seq peaks from the DA- specific ATAC-seq repertoire (GREAT). (**f**) Sox3 binding motif frequently found at loci associated with DA-specific intergenic ATAC-seq peaks (RSAT). (**g**) Pathway analysis (Wikipathway 2016, Enrichr) performed on the DA-specific ATAC-seq peaks associated with promoters.

We used ATAC-seq technology to identify potential regulatory regions of the chromatin active in DA neurons. We distinguished 45,402 ATAC-seq peaks present simultaneously in the 3 ATAC-seq datasets obtained from mesencephalic DA neurons (Fig. 4a), distributed within promoters (19%), intragenic (40%, comprising exons and introns) and intergenic (38%) loci (Fig. 4b). In comparison, we found 18,658 ATAC-seq peaks representing open chromatin regions in both datasets originating from hindbrain 5-HT neurons. In contrast to the DA repertoire of regulatory regions, the majority of 5-HT ATAC-seq peaks were first associated with promoters (37%), then with intragenic (35%) and intergenic (25%) loci (Fig. 4a,b).

Among both these lists of potentially active regulatory regions, 16,856 of them were found in all of the DA and 5-HT datasets, and were considered as common (Fig. 4c). Interestingly, we identified 17,616 ATAC-seq peaks present in the 3 DA datasets but absent in the 5-HT datasets, indicating that 39% of the DA open chromatin regions were specific to this neuronal subtype (Fig. 4c). Conversely, only 513 ATAC-seq peaks were detected in all the 5-HT data sets and not within the DA peaks, constituting a small fraction of 3% of the 5-HT repertoire that was cell-specific. This correlated with the transcriptomic data (Supplementary Fig. S2d) that suggested that the 5-HT cell populations expressed common genes with DA neurons.

Gene Ontology (GO) enrichment analysis performed on genes associated with DA-specific intragenic ATAC-seq peaks, i.e. only detected in the 3 DA datasets, revealed numerous terms linked to biological processes involved in central nervous system development and maturation (Fig. 4d), including axon guidance and notably “dopaminergic neuron axon guidance” (p-value = 4.363 × 10^−10^; Fig. 4d). We then focused on intergenic ATAC-seq peaks that include regulatory regions such as distal enhancers and completed a similar analysis on adjacent genes relative to DA-specific intergenic ATAC-seq peaks. We found a significant gene enrichment in biological processes involving neuronal development, including “midbrain development” (p-value = 1.95 × 10^−12^; Fig. 4e), confirming the cell specificity of these ATAC-seq peaks. In addition, we discovered that these intergenic open chromatin regions were significantly enriched with a DNA-binding motif associated with the transcription factor Sox3 (e-value = 1.2 × 10^−30^), which has notably been associated with neurogenesis^47^ (Fig. 4f). Finally, a pathway analysis demonstrated that the term “Dopaminergic neurogenesis” was significantly enriched (p-value = 0.0007433) in genes whose promoters were associated with DA-specific ATAC-seq peaks (Fig. 4g). Altogether, these data not only indicate that we identified the mesencephalic DA map of open chromatin regions, but also substantiate the cell-specificity of this repertoire obtained by ATAC-seq.

**Figure 5:**
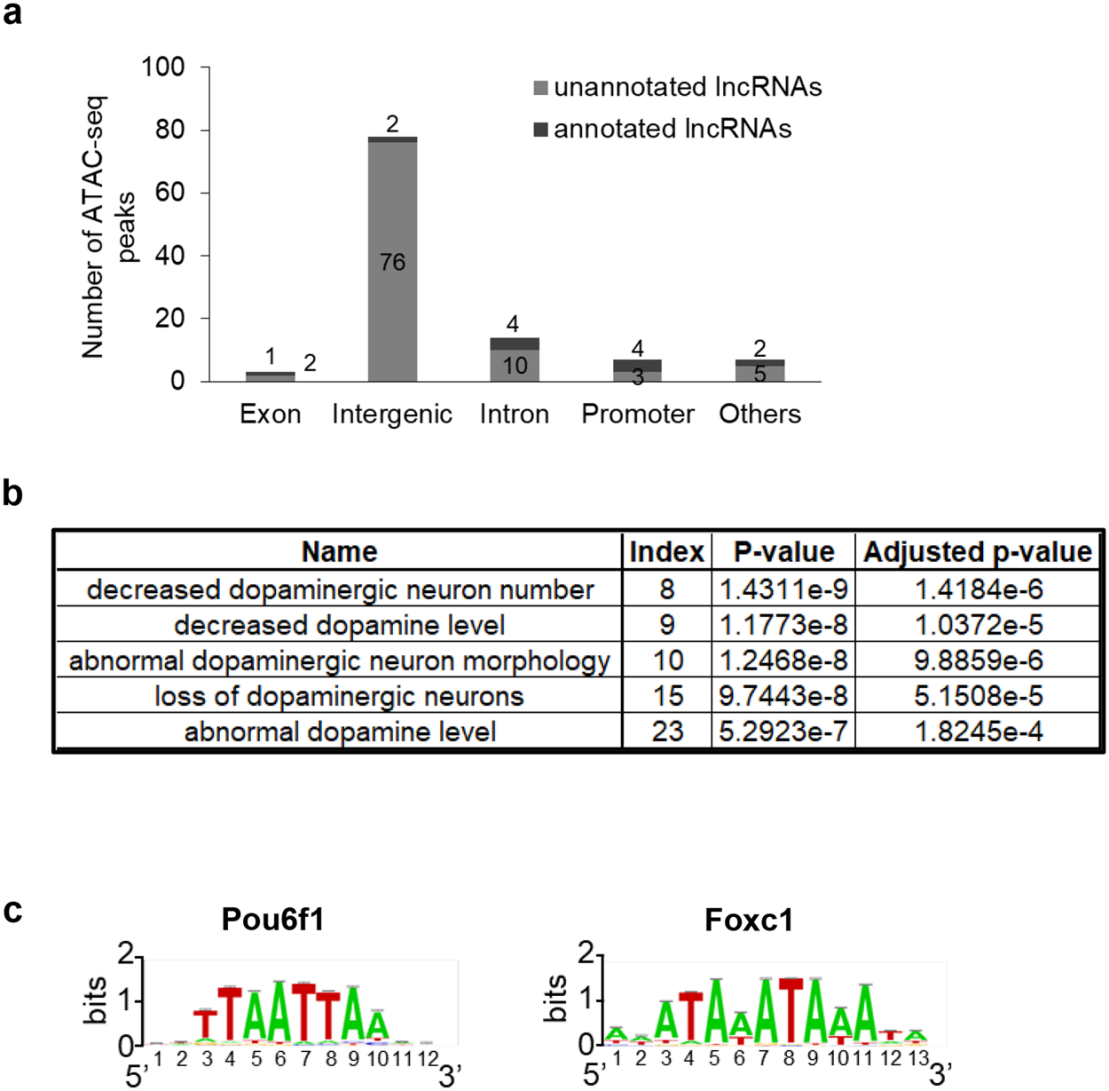
Analysis of DA-specific ATAC-seq peaks coinciding with promoters of DA lncRNAs. (**a**) Number of DA-specific ATAC-seq peaks associated with DA lncRNAs, depending on their genomic loci. Dark grey, lncRNAs annotated in Ensembl; light grey, lncRNAs unannotated. (**b**) MGI Phenotype ontology enrichment analysis performed on these DA-specific ATAC-seq peaks associated with DA lncRNAs (GREAT). (**c**) Binding motifs found at loci associated with DA-specific ATAC-seq peaks associated with DA lncRNAs.

By cross-analysing both DA repertoires of lncRNAs and open chromatin regions, we identified 109 DA-specific ATAC-seq peaks overlapping the TSS of lncRNAs (Fig. 5a). Interestingly, 96 out of these 109 lncRNAs were identified for the first time (unannotated) and the majority of them were intergenic. Using Mouse Genome Informatics (MGI) Phenotype ontology, we found that numerous terms describing phenotypes linked to DA neurons were enriched with genes neighbouring these specific lncRNAs (Fig. 5b). *De novo* motif discovery with DA-specific promoters (Fig. 5c) suggested that they are enriched for the motifs bound by Pou6f1 (e-value = 9.5 × 10^−3^), a transcription factor expressed in post-mitotic neurons^48^, and Foxc1 (e-value = 1.5 × 10^−4^) whose expression has been shown to be downregulated in the midbrain DA neurons from patients with Parkinson’s Disease^49^.

### Expression analysis of lncRNAs in a primary culture of E14.5 ventral mesencephalons

**Figure 6:**
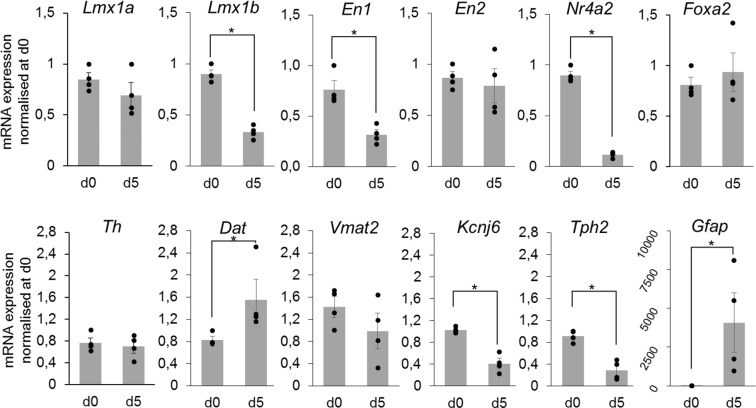
mRNA expression of DA marker genes of DA progenitors (*Lmx1a*, *Lmx1b*, *En1*, *En2*, *Nr4a2*, *Foxa2*), differentiated DA neurons (*Th*, *Dat*, *Vmat2*, *Kcnj6*), 5-HT neurons (*Tph2*) and astrocytes (*Gfap*) in primary cultures from E14,5 ventral mesencephalons at day 0 and after 5 days. Grey columns represent the mean value of 4 independent culture experiments depicted by black circles. mRNA expression was normalized relatively to *Tbp* mRNA expression. Values represented correspond to the mean value of the 3 replicates from each experiment, normalised to the value of 1 reference experiment at day 0. Error bars show standard error of the mean. *p-value ≤ 0,05.

**Figure 7:**
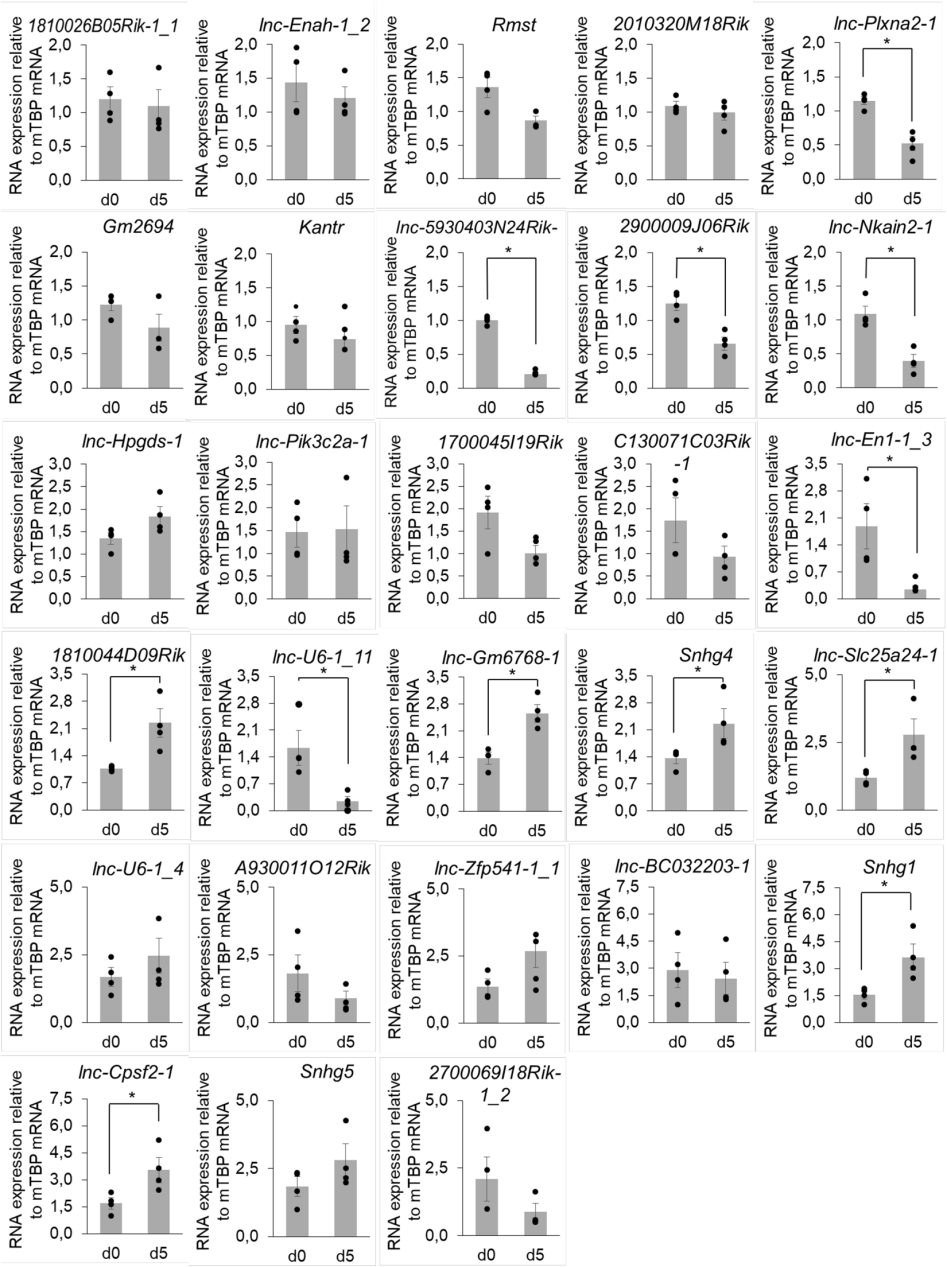
RNA expression of selected lncRNAs in primary cultures from E14,5 ventral mesencephalons at day 0 and after 5 days. Grey columns represent the mean value of 4 independent culture experiments depicted by black circles. mRNA expression was normalized relatively to *Tbp* mRNA expression. Values represented corresponds to the mean value of the 3 replicates from each experiment, normalised to the value of 1 reference experiment at day 0. Error bars show standard error of the mean. *p-value ≤ 0,05.

After dissection and dissociation of E14.5 ventral mesencephalons, we cultured cells for 5 days (d5) in order to study selected lncRNAs from the DA repertoire. First, using RT-qPCR to assess marker genes of the DA lineage (Fig. 6), we observed that the cell population obtained presented DA progenitors at d0 and d5, with some markers such as *Lmx1b*, *En1* and *Nr4a2* showing a decreased expression at d5. Markers of differentiated DA neurons were also expressed at both time points, such as *Th*, *Dat*, *Vmat2* and *Kcnj6*. Interestingly, the increase in *Dat* expression suggested some degree of DA neurons maturation in culture. Moreover, *Kcnj6* has been shown to be expressed more abundantly in DA neurons from the SNpc than the VTA^50^, and thus its decreased expression from d0 to d5 implied that at least a fraction of DA neurons present in the culture potentially displayed a VTA identity. Expression of the 5-HT marker gene *Tph2* decreased from d0 to d5, however as expected we noticed a massive increase in *Gfap* expression, reflecting proliferation of astrocytes. We then evaluated whether we could detect lncRNAs from the DA repertoire in this system. Selection of lncRNAs was based on literature curation searching for: (i) implication of their adjacent coding-genes, or themselves, in neuronal development and differentiation, ideally in the DA lineage; (ii) potential involvement in brain pathology. Examples of genomic organization of selected lncRNAs is presented in Supplementary Fig. S5. We analysed expression of 28 lncRNAs (Table 1 and Fig. 7), most of which were stably expressed from d0 to d5. Some displayed an increase in expression, such as *Snhg1*, which has been shown to play a role in cell proliferation in cancer^51–53^, and has been associated with patients suffering from Parkinson’s Disease^35^. Others, including the novel lncRNA *lnc-En1-1_3* whose closest gene is En1, decreased from d0 to d5.

**Table 1:** Brief description of the selected lncRNAs analysed in Fig. 7.

	exons	locus	category	closest coding genes		overlapped gene
				downstream	upstream	
**1810026B05Rik-1_1**	5	chr7:80688957-80703006	intergenic	Chd2	A830073O21Rik	—
lnc-Enah-1_2	2	chr1:183950276-183959960	divergent	Srp9	Enah	—
Rmst	4	chr10:91618180-91628236	intergenic	Gm16484	Nedd1	—
**2010320M18Rik**	1	chr8:73300708-73301478	divergent	Mast3	Pik3r2	—
**lnc-Plxna2-1**	1	chr1:196444409-196445898	divergent	Camk1g	Plxna2	—
Gm2694	4	chr8:89996573-90049469	divergent	4933402J07Rik	Cbln1	—
Kantr	3	chrX:148729370-148762038	intergenic	Kdm5c	Tspyl2	—
**lnc-5930403N24Rik-1**	1	chr10:36859252-36861073	overlapping antisense	Marcks	Lama4	5930403N24Rik
2900009J06Rik	2	chr1:129650242-129670663	overlapping antisense	Tmem163	Ccnt2	Acmsd
**lnc-Nkain2-1**	1	chr10:32609904-32611996	overlapping antisense	Rnf217	Trdn	Nkain2
lnc-Hpgds-1	1	chr6:65100926-65101303	intergenic	C130060K24Rik	Hpgds	—
**lnc-Pik3c2a-1**	1	chr7:123587063-123587841	divergent	Nucb2	Pik3c2a	—
1700045I19Rik	1	chrX:160198072-160199261	intergenic	Ap1s2	Grpr	—
**C130071C03Rik-1**	1	chr13:83867069-83868718	intergenic	Tmen161b	Mef2c	—
**lnc-En1-1_3**	8	chr1:122518213-122586980	intergenic	Insig2	En1	—
**1810044D09Rik**	2	chr6:91390997-91391746	intergenic	Chchd4	Wnt7a	—
**lnc-U6-1_11**	6	chr15:89875438-89905660	intergenic	Alg10b	Syt10	—
**lnc-Gm6768-1**	1	chr12:120591340-120591768	intergenic	Macc1	Itgb8	—
Snhg4	4	chr18:35713064-35717970	intergenic	Matr3	Gm5239	—
**lnc-Slc25a24-1**	2	chr3:108923456-108925959	divergent	4930443G12Rik	Slc25a24	—
**lnc-U6-1_4**	1	chr1:4677948-4679320	intergenic	Sox17	Mrpl15	—
**A930011O12Rik**	2	Chr14:65208662-65212786	intergenic	Kif13b	Msra	—
**lnc-Zfp541-1_1**	1	chr7:16633660-16634413	intergenic	Zfp541-1	Gltscr1	—
lnc-BC032203-1	1	chr17:46993166-46994043	intergenic	BC032203	A330017A19Rik	—
Snhg1	10	chr19:8797802-8800934	divergent	Wdr74	Slc3a2	—
**lnc-Cpsf2-1**	3	chr12:103206308-103213872	divergent	Atxn3	Cpsf2	—
Snhg5-1	5	chr9:88415894-88417721	intergenic	Syncrip	Zfp949	—
**2700069I18Rik-1_2**	1	chr3:5219240-5219623	overlapping antisense	Gm10748	Pxmp3	Zfhx4

For each lncRNA, the number of exons, locus, category, closest coding-genes and overlapped genes are provided. In bold are represented the DA-specific lncRNAs relatively to the 5-HT repertoire.

## Discussion

In this study, we identified and characterized the DA repertoires of lncRNA loci and open chromatin regions from ventral mesencephalons at E14.5. We found 1,294 lncRNAs expressed in DA neurons, among which 939 had not been previously described. Most of these transcripts were intergenic or divergent. As a comparison, we also identified 1,293 lncRNAs expressed in hindbrain 5-HT neurons, comprising 806 novel transcripts. Regarding their position relative to the closest gene, 5-HT lncRNAs were predominantly intergenic and overlapping antisense. Moreover, both repertoires reflected the two distinct populations since only 165 lncRNAs were found in common. In parallel, ATAC-seq analysis allowed for the identification of 45,402 open chromatin regions in E14.5 DA neurons, more than twice the number of ATAC-seq peaks observed in 5-HT neurons (18,658). These putative active regulatory regions were distributed within intergenic, exonic, and intronic loci, as well as within promoter regions. Comparing regions of open chromatin from DA and 5–HT neurons, we observed that more than a third of the DA repertoire were not found in the 5-HT neurons, whereas most of the 5-HT ATAC-seq peaks were also mapped in DA neurons. We overlapped the DA repertoires of lncRNAs and open chromatin regions and identified specific regions mostly associated with novel DA lncRNAs. Finally we selected lncRNAs expressed in the DA datasets and analysed their expression in a primary culture of ventral mesencephalons.

Consistent with data from the literature, our study highlights the high degree of cell-specificity of both lncRNAs repertoire and map of open chromatin, that actually represent more accurate molecular signatures associated with cellular subtypes than protein-coding genes (Fig. 3)^15–19^. Regarding lncRNAs, we indeed observed that the majority of the lncRNAs expressed in DA neurons were described for the first time, and the comparison with lncRNAs identified from 5-HT neurons highlighted even more this cell-specificity as only a small fraction of transcripts were expressed in both neuronal subtypes. However, in contrast with many studies^19,54,55^, we did not eliminate transcripts carrying a single exon in our identification criteria but retained monoexonic transcripts with a clear ATAC-seq signal overlapping their TSS. Example of a novel monoexonic transcript is illustrated in Supplementary Fig. S5d. Using such stringent criteria, we cannot rule out that some monoexonic lncRNAs are missing in the repertoires due to the sequencing under-representation of transcript 5′ ends (Supplementary Fig. S1). This technology bias is more important for long transcripts. The average length of monoexonic transcripts is of 918 nt and 745 nt in the DA and 5-HT repertoires respectively, consistent with the size range where the sequencing bias is minimal (Supplementary Fig. S1). The resulting repertoire contained 73.1% of monoexonic transcripts among the novel lncRNAs identified (and only 16.9% among the annotated lncRNAs). Therefore we cannot exclude that the cell-specificity that we observed was in part due to a bias associated with the lack of annotation of single exon lncRNAs. Nevertheless, this bias does not imply that these monoexonic lncRNAs were not specifically expressed in DA neurons, and the fact that we found a majority of cell-specific transcripts comparing DA and 5-HT lncRNAs using the same criteria to generate both repertoires, indicated that both constitute molecular signatures of the neuronal subtypes they have been generated from. Moreover, for the lncRNAs identification process and further cell-specificity analyses, we focused on transcripts expression, rather than gene expression. Thus, we noticed that several transcripts could be detected at the same locus, representing isoforms of the same gene, and decided to only consider the most expressed transcript. Using this strategy, we took into account not only the transcription process, but also the splicing process that is still poorly studied regarding lncRNAs. This way, we have been able to identify cell-specific isoforms of lncRNAs, resulting in a number of cell-specific lncRNAs more precise and higher than if we had chosen an identification process based on genes.

In order to select lncRNAs actively transcribed, we used ATAC-seq data to identify TSS of our candidate transcripts. However, we observed numerous multiexonic transcripts that were expressed within our criteria, but still did not harbour an ATAC-seq peak at their TSS. Again, a possible explanation lies in the sequencing of polyadenylated RNA that often results in a decrement in mapped reads towards the first exons. It is therefore possible that some of the expressed lncRNAs that were not displaying an ATAC-seq peak at their putative TSS were in fact not integrally sequenced. It is also possible that these lncRNAs could be detected with RNA-seq, but that there was no permissive region detectable at their promoter by ATAC-seq. This could be explained by very weak transcriptional activity producing stable transcripts and/or active transcription in a small subset of cells. To circumvent this issue, single cell deep RNA-seq and ATAC-seq for the study of lncRNAs is essential, even though the development of these techniques at this scale is still at its beginning.

We generated maps of open chromatin from DA and 5-HT neurons and observed that the DA datasets displayed far more putative regulatory regions than the 5-HT datasets. Such difference between cell types has already been described^56^ and is potentially intrinsically associated with the nature of the cells studied. However, the percentage of ATAC-seq peaks specific to DA neurons was strikingly higher than the peaks only present in the 5-HT neurons. While this observation demonstrates consistency between DA repertoires of lncRNAs and open chromatin regions that both display an important cell-specificity, it shows quite a difference within the 5-HT repertoires. Indeed, many lncRNAs but only 3% of the open chromatin regions were specific to 5-HT neurons. The weak specificity level of ATAC-seq peaks compared to the DA datasets could however reflect the fact that the cell population extracted from E14.5 r1-3 rhombomeres also expressed DA marker genes. Also, because of the reduced numbers of ATAC-seq peaks in 5-HT neurons compared to DA neurons, we cannot rule out that the number of monoexonic transcripts that are intergenic was underestimated in 5-HT neurons (139 *versus* 384 in the DA repertoire, see material and methods). Indeed, we chose to discard from our analysis monoexonic transcripts that do not harbour an ATAC-seq peak at their putative TSS in our analysis. However, the difference between the DA and 5-HT ATAC-seq datasets is principally due to a preferential loss of intergenic and intronic peaks (respectively 3.6 and 3.1 times less in 5-HT neurons, Fig. 4a). Therefore, it is possible that some intergenic lncRNAs displaying a single exon in the 5-HT repertoire have been eliminated from our analysis.

Importantly, Genome Wide Association studies have allowed identification of many SNPs in human pathologies that are associated with non-coding regions of the genome^29,30,57^, suggesting that numerous risk factors linked to diseases could alter the function of lncRNAs or enhancer regions. Our work substantiates the increasing literature showing that lncRNAs and open chromatin regions constitute very specific molecular signatures, and strengthens the need to study these elements in distinct cellular subtypes, especially in the context of human pathologies that are associated with dysfunction of specific cells, such as cancers, Diabetes or Parkinson’s Disease. Interestingly, 44 Parkinson’s disease risk loci have been identified from meta-analysis of genome wide association studies^57,58^. Using synteny analysis, we found 8 lncRNAs of the DA repertoire located in the mouse syntenic regions corresponding to genomic areas of the Parkinson’s disease human risk loci, (Supplementary Table S2). Among these lncRNAs, 6 were unannotated and 5 of them were specifically found in DA neurons. Although found in both DA and 5-HT neurons, 2900009J06Rik-1, one of the candidate lncRNAs we studied, is significantly more expressed in ventral mesencephalons than in rhombencephalons of E14.5 embryos (Supplementary Fig. S5). Overall, this highlights the cell-specificity of the lncRNAs potentially linked to Parkinson’s Disease. Discovery of cell-specific regulatory lncRNAs or regulatory DNA sequences might therefore provide new clues towards a better comprehension of human diseases but also advancements in the search of therapeutic targets.

## Material and Methods

### Animals

All procedures were conducted in compliance with the European and French legislations (EU directive 2010/63/UE), and were approved by the “Direction Départementale de la Protection des Populations” under accreditation number A75-13-19.

To purify dopaminergic (DA) neurons by fluorescence-activated cell sorting (FACS) prior to RNA-seq and ATAC-seq, we used TH-GFP mice, in which GFP is expressed under the control of the *Th* promoter^41^. This transgenic line, maintained on C57BL/6J background, was generously given by H. Okano. To isolate serotonergic (5-HT) neurons by FACS, we were generously given by C. Parras Mash1-CRE × ROSA YFP mice, in which YFP is expressed in 5-HT neurons^46^. Mice had *ad libitum* access to food and water, and were housed in cages containing up to 5 animals under temperature-controlled conditions and maintained on a 12/12 hours light/dark cycle.

To obtain E14.5 embryos, males from these transgenic lines were mated with Swiss wild-type females overnight, and pregnancies confirmed the next morning by inspection of the vaginal plug, defining embryonic day 0.5.

For primary cell culture experiments, we used E14.5 embryos of Swiss wild-type mice purchased from Charles River, France.

### Tissue collection

To obtain DA neurons, 5–15 ventral mesencephalons from E14.5 embryos were dissected for each experiment. Regarding 5-HT neurons, 4–8 regions containing rhombomeres r1, r2 and r3 of the hindbrain were carefully removed from the embryonic brains for each experiment. After removing the meninges, tissue was collected in ice-cold HBSS 1X until dissociation.

### Fluorescence-activated cell sorting (FACS)

Tissue was mechanically dissociated into a single cell suspension in Neurobasal medium with B27 (Life Technologies) and kept at 4 °C until FACS purification. Cell suspensions were filtered with a 50 μm filter and then processed by FACS for selection of DA GFP^+^ or 5′HT YFP^+^ cells. FACS was performed on an INFLUX 500 cell sorter. Dead cells were excluded by addition of propidium iodide. Cell suspensions from ventral mesencephalons or hindbrain r1-3 of wild-type mice were used to adjust background fluorescence. For RNA-seq, single cells were collected in Lysis Buffer (RNeasy Micro Kit Qiagen) with 1% of β-mercapto-ethanol and immediately kept in dry ice at −80 °C until RNA extraction. For ATAC-seq or isolated cells culture on GFP^+^ or YFP^+^ cells, single cells were collected in Neurobasal medium with B27 supplement (Life Technologies), 2% FBS and kept at 4 °C.

### Immunofluorescence

An average of 8000 cells sorted by FACS (GFP^+^/GFP^−^ and YFP^+^/YFP^−^) were independently plated on Poly-D-lysine hydrobromide (Sigma) coated Labteck, cultured for 90 min in Neurobasal medium with B27 supplement and 20% FBS. Cells that attached were fixed with 4% paraformaldehyde in PBS for 10 minutes, followed by successive washes with PBS and PBS-Tween (1X, 0.1% Tween). After incubation in blocking solution for 1 h (PBS-Tween 10% goat serum), cells were immunolabelled overnight at 4 °C using the following antibodies: mouse anti-TH (1:400; Millipore MAB318), rabbit anti-GFP-YFP (1:750; Megaprob A11122), rabbit anti-TPH2 (1:500; Novus Biologicals). The next day, samples were washed, then incubated during 1 h at room temperature with the following secondary antibodies: goat anti-rabbit Alexa 555 (1:1000; Invitrogen A-21428), goat anti-rabbit Alexa 488 (1:1000; Invitrogen A-11008), goat anti-mouse Alexa 555 (1:1000; Life Technologies A21425). Nuclei were labelled with Hoechst. All images were collected on a Leica microscope.

### Primary cell culture from E14.5 ventral mesencephalons

Dissociation of E14.5 ventral mesencephalons was performed by alternating mechanical dissociation and 10 min decantation steps 4 times. Dissociated cells from E14.5 ventral mesencephalons were cultured in 12 well-plates previously coated with Neurobasal medium complemented with 1% ECM (Sigma-Aldrich), Fibronectin from bovine plasma (1:250, Sigma-Aldrich) and 1% Penicilin/streptomycin (Life Technologies). Around 1 ventral mesencephalon was used for each well. Cells were incubated for 5 days at 37 °C, 5% CO_2_ with Neurobasal medium complemented with B27 + vitamin A supplement (1:50; Life Technologies), 1% L-glutamine (Life Technologies), 1% Penicilin/streptomycin, 20 mg/mL GDNF (Peprotech), 1 mM AMPc (Sigma-Aldrich) and 20% FBS (Helvetica Health Care). Twenty mg/mL of freshly made GDNF was added at days 2 and 4. Four independent experiments were conducted. For each experiment, dissociated cells were distributed within 3 wells at day 0 and cultured independently until collection at day 5 for RT-qPCR.

### RNA extraction

Total RNA was extracted from DA or 5-HT neurons using an RNeasy Microkit (Qiagen) following manufacturer’s instructions. RNA was treated with DNAse I (Qiagen) for 20 minutes at room temperature to prevent genomic DNA contamination. For RT-qPCR, RNA concentrations were determined by spectrophotometry (Nanodrop 2000c, THERMO Scientific). For RNA-seq, a High Sensitivity RNA ScreenTAPE analyzer (Agilent Technologies) was used to assess RNA concentrations as well as the RNA integrity number (RIN) to verify RNA quality for all tested samples. RNA was stored at −80 °C until reverse transcription or RNA-seq.

### Real time quantitative RT-PCR (RT-qPCR)

Up to 500 ng RNA was used to generate a first cDNA strand (Superscript II reverse transcriptase, THERMO Fisher Scientific) with random hexamers as indicated by the manufacturer. qPCR experiments were realized on the Light Cycler 96 or 1536 real-time PCR system (Roche); with SYBER green detection. The comparative method of relative quantification (2^−ΔΔ*CT*^) was used to calculate the expression levels of each target gene and mouse *Tbp* mRNA was used to normalize the expression of all samples. The list of primers used is provided in the Supplementary table S3.

### RNA-sequencing (RNA-seq)

Three independent FACS experiments were achieved for each neuronal subtype, providing 34,110 to 82,678 GFP^+^ cells for DA neurons and 9,207 to 11,107 YFP^+^ cells for 5-HT neurons. One ng of total RNA was used for RNA-seq. For FACS-purified samples of DA neurons, stranded library was prepared using TotalScript RNA sequencing kit (Epicentre) following manufacturer’s recommendation. For purified samples of 5-HT neurons, non-stranded library were prepared using SMART-Seq v4 Ultra Low Input RNA Kit for Sequencing (Clontech) following manufacturer’s recommendation. 3 DA and 3 5-HT libraries were sequenced using NextSeq500 HighOutputKit v2 (300cycles) cartridge (FC-404-2004 Illumina).

### ATAC-sequencing (ATAC-seq)

Three independent FACS experiments were achieved for DA neurons analysis, providing 50,000 to 87,500 GFP^+^ cells, and 2 independent FACS experiments were performed for 5-HT neurons analysis, providing respectively 11,448 and 19,407 YFP^+^ cells. Sorted cells by FACS were collected in Neurobasal medium with B27 supplement (Life Technologies), 2% FBS and kept at 4 °C until ATAC-Seq. Cells were centrifuged at 500 g, at 4 °C during 20 min. Cells were resuspended in 25 μl of lysis buffer (10 mM Tris-HCl pH 7.4, 10 mM NaCl, 3 mM MgCl_2_, 0.1% IGEPAL CA-630) during 10 min at 4 °C. Then supernatant was taken out after a centrifugation at 500 g, at 4 °C during 30 min. For transposase reaction, the pellet was resuspended in 25 μl of 12.5 μl 2x TN buffer; 2 μl of Tn5; 10.5 μl d’H2O and incubated at 37 °C for 1 h. Then 5 μl of clean-up buffer (900 mM NaCl, 300 mM EDTA, 5% SDS) were added with 2 μl of 5% SDS and 2 μl of Proteinase K, and cells were incubated for 30 min at 40 °C. Samples were then cleaned with two SPRI clean up (Agencourt © AMPure ©XP), with 68 μl of SPRI beads, eluted in 13 μl of buffer EB (Qiagen Cat No./ID: 19086). Extracted DNA concentration was measured by ScreenTAPE analyzer (Agilent Technologies). To generate libraries, PCR reactions were performed using the kapa PCR mix (Kapa biosystem) with 12.5 μl Kapa, 1 μl primers and 11.5 μl of sample, with the NextEra primers (1 μl /primer). PCR conditions were performed as described: 98 °C during 2 min and then 9 cycles of 98 °C during 20 s, 63 °C during 30 s, 72 °C during 1 min. Then, a new SPRI clean-up was made to do a size cut off of amplified PCR products and after that, DNA concentration was measured by ScreenTAPE analyzer (Agilent Technologies). Finally, a second PCR was performed with the same conditions as the first one and a last SPRI clean-up was made and library of tagged open chromatin was ready to be sequenced. Libraries were sequenced, with 75 bp paired-end reads, on an Illumina NextSeq500 plateform,

### Bioinformatics

#### RNA-seq data processing

Raw sequencing data was quality-controlled with the FastQC program. Adapter sequences were removed by Cutadapt. Low quality reads were trimmed or removed using Trimmomatic (minimum length: 40 bp). Reads were aligned to the mouse reference genome (build mm9) with the TopHat2 tool^59^ (option for no multihits) and mapping results were quality-checked using RNA-SeQC. Normalization and differential analysis were performed with the DESeq2 package.

#### ATAC-seq data processing

Steps for quality control were identical to those used for RNA-seq data treatment (Trimmomatic, FastQC). Reads with a length below 100 bp have been removed in further analysis. Paired-end reads were mapped to the mouse genome (build mm9) with Bowtie2. Duplicate reads were discarded with the Picard tools. Peaks were called using the MACS2 program with the option callpeak. Individual peaks separated by less than 100 bp were merged with BEDOPS and features annotations were obtained from the HOMER mm9 database. We mapped 52,862; 93,056 and 94,352 peaks from the 3 DA datasets, and 38,772 and 22,880 peaks from the 2 5-HT datasets. We extracted the ATAC-seq peaks present simultaneously in the 3 ATAC-seq datasets obtained from DA neurons (n = 45,402) and the ATAC-seq peaks in both datasets originating from 5-HT neurons ( = 18,658), as shown in Fig. 4a. To analyse the number of specific or common ATAC-seq peaks between the DA and 5-HT datasets, we selected the DA-specific peaks as peaks present in the 3 DA datasets but absent in both 5-HT datasets; the 5-HT-specific peaks as peaks present in both 5-HT datasets but absent in the 3 DA datasets; and the common ATAc-seq peaks between DA and 5-HT neurons as peaks present in the 5 datasets. We therefore excluded from this stringent analysis the peaks found in the 3 DA datasets but only in one 5-HT dataset (n = 10930), as well as the peaks found in both 5-HT datasets but only in one or two DA datasets (n = 1289).

Data from ATAC-seq and RNA-seq results were intersected based on overlaps between a given ATAC peak and the first/last nucleotide of a TSS/TTS, respectively.

#### Construction of lncRNA catalogues

Transcriptomes were assembled using the Cufflinks/Cuffmerge suite, guided by the GENCODE GTF mm9 annotation file. Quantification and normalization at the gene (XLOC) and transcript (TCONS) levels were performed with Cuffquant/Cuffnorm. A consolidated result file containing transcript attributes (e.g., exon number, Cufflinks class-code), FPKM values, intersection with ATAC peaks as defined above, and GENCODE annotations, was then produced. Annotated lncRNAs were selected from TCONS entries whose Cufflinks class-codes were different from ‘−’ (Unknown, intergenic transcript) and ‘x’ (Exonic overlap with reference on the opposite strand) and whose annotation contained one of the following biotype: “lncRNA”, “antisense”, “non_coding”. Potential novel lncRNAs were identified from TCONS entries characterized by Cufflinks class-codes ‘−’ or ‘x’. For both classes, the following criteria were used: length > 200 bp and FPKM ≥ 1 in at least 1 sample out of 3 replicates.

The closest protein-coding gene for each lncRNA was identified using the tool ‘bedtools closest’. Coding potential of transcripts was assessed using CPAT^42^ (cut-off: 0.44). Those information were included in the repertoire consolidated file. Moreover, lncRNAs located at a distance lower than 1 kb from a known gene on the same strand (or whose strand was undetermined), were eliminated.

Due to technical reasons, the libraries of 5-HT neuron RNA-seq experiments were not performed in a stranded-specific manner, resulting in the inability to infer the strand for unannotated monoexonic transcripts. Regarding transcripts displaying multiple exons, assignment of the strand was performed by identification of consensus splice sites by the TopHat2 tool. Since our strategy was to discard transcripts lying within 1 kb from a protein-coding gene on the same strand, this led us to discard all unannotated monoexonic transcripts located at less than 1 kb from a protein-coding gene for the 5-HT repertoire, leaving mostly intergenic lncRNAs (i.e. located at a distance superior to 1 kb from a protein-coding gene). Thus, for comparative analyses of specificity between DA and 5-HT repertoires, we have compared lncRNAs presenting the same characteristics in both repertoires, i.e. we excluded the monoexonic transcripts located at less than 1 kb of a protein-coding gene from the DA repertoire. This way we avoided the introduction of a biased estimation of cell specificity. Correspondences between lncRNAs of the two cell type repertoires were defined with the following strategy. A reciprocal intersection between transcript coordinates of each repertoire was computed with a threshold equal to 90% of sequence length in common; the number of exons of the pairs thus defined had to be identical; coordinates for each pair of exons had to differ from no more than 50 bp for internal exons and 500 bp for outermost exons. Then manual curation has been performed to ensure the strength of the correspondences. lncRNA labelled as “Specific gene” do not have counterpart in the repertoire of the other cell type. LncRNAs expressed as distinct isoforms in each cell type belong to the category called “same gene specific isoform”. LncRNAs expressed as the same isoform in both repertoires are labelled as “same gene same isoform”, and are non-specific.

### Gene Ontology (GO), Pathway enrichment analysis and search for transcription factors motifs

To perform GO and Pathway enrichment analysis on a list of genes, Enrichr was exploited, using GO Biological process, Panther 2016, and Wikipathway 2016 databases^60,61^. To perform these analyses on non-coding regions, we used GREAT that analyses the annotations of the nearby genes, using GO biological processes and MGI phenotype ontology databases^62^. To search for transcription factors-associated motifs, RSAT was used^63^.

### Statistics

Statistical analyses to assess differences in gene expression from day 0 to day 5 of cell cultures were conducted using two-tailed Mann Whitney *U*-tests (GraphPad Prism 6). For each gene, values were normalised to the mean of the 3 replicates of 1 experiment at day 0. Values represented therefore correspond to the mean value of the 3 replicates from each experiment, normalised at day 0.

One-tailed Mann Whitney *U*-tests were used to compare lncRNA expression between ventral mesencephalons and r1-3 rhombomeres. Mann Whitney U-tests and p-values are gathered in Supplementary Tables S4.

For GO and Pathway enrichment analysis on a list of genes with Enrichr, a Fisher exact test was used. A Binomial test over genomic regions was performed for GO biological processes and MGI phenotype ontology analyses using GREAT.

## Supplementary information

Supplementary material

## Data Availability

The GEO accession number for RNA-Seq and ATAC-Seq reported in this paper is: GSE108917.
