# Supplementary material for: Long non-coding RNA repertoire and open chromatin regions constitute midbrain dopaminergic neuron - specific molecular signatures
Source: Sci Rep. 2019 Feb 5;9:1409. doi: 10.1038/s41598-018-37872-1 (PMC6363776; doi:10.1038/s41598-018-37872-1)
Supplement: Supplementary file 1 — Supplementary material [file 41598_2018_37872_MOESM1_ESM.pdf]

Long non-coding RNA repertoire and open chromatin regions constitute midbrain dopaminergic neuron - specific molecular signatures.

Gendron J., Colace-Sauty C., Beaume N. Cartonnet H., Guegan J., Ulveling D., Pardanaud-Glavieux C., Moszer I. , Cheval H. and Ravassard P.

SUPPLEMENTARY MATERIAL

SUPPLEMENTARY TABLES

Supplementary Table S1. Top 100 most expressed lncRNAs from the DA repertoire. The transcript ID, locus, number of exons, strand, category, as well as the official annotation, if existing, of each lncRNA are provided. If unannotated, the lncRNA is called lnc-name_of_the_closest_coding_gene

Supplementary Table S2. lncRNAs from the DA repertoire located in syntenic mouse regions of Parkinson's Disease-associated risk loci.
For each locus, we selected a 400 kbp region centered on the most significant single nucleotide polymorphism (SNP) and performed synteny analysis to search for the corresponding mouse chromosomal region. Precisely, syntenic regions were identified using pairwise whole genome alignments (*net.axt* files) of hg19 vs mm9 from UCSC. Then bedtools was used to intersect our region of interest with these *net.axt* files, followed by manual curation.
LncRNAs from the DA repertoire found in the mouse syntenic loci are represented in the table, not yet annotated lncRNAs are in bold, and DA-specific lncRNAs (compared to the 5-HT repertoire) are contained in a grey cell.

Supplementary Table S3. List of primers used for quantitative RT-PCR.

Supplementary Table S4. Mann Whitney U tests and p values regarding expression of marker genes (a) and candidate lncRNAs (b) in primary cultures from E14,5 ventral mesencephalons at day 0 compared to day 5 (Fig. 6 and 7), as well as selected lncRNAs expression in ventral mesencephalons compared to rhombomeres r1-3 of E14,5 mice embryos (c).

Supplementary Table S1

tracking ID	locus	exons	strand	transcript ID	gene name	category
TCONS_00070610	chr19:5801943-5802640	2	-	ENSMUST00000173523.1	Malat1	Intergenic
TCONS_00032077	chr12:110784655-110809932	5	+	ENSMUST00000143847.1	Meg3	Intergenic
TCONS_00060067	chr17:39979943-39985776	1	+	ENSMUST00000062783.8	Rn45s	Intergenic
TCONS_00003581	chr1:162964553-162968711	11	+	ENSMUST00000162558.1	Gas5	overlapping antisense
TCONS_00034453	chr12:103206308-103213872	3	-	-	lnc-Cpsf2-1	divergent
TCONS_00031928	chr12:106727948-106731745	2	+	-	lnc-Bdkrb2-1	Intergenic
TCONS_00141055	chr8:89996573-90049469	4	+	ENSMUST00000123808.1	Gm2694	divergent
TCONS_00004195	chr1:180229446-180230507	2	+	-	lnc-B230369F24Rik-1	Intergenic
TCONS_00115668	chr5:112641613-112649603	3	-	ENSMUST00000146058.2	Miat	Intergenic
TCONS_00047349	chr15:62812392-62813249	2	+	-	lnc-SNORA17-1_6	Intergenic
TCONS_00102766	chr4:41585177-41587357	2	-	ENSMUST00000127306.1	Enho	overlapping antisense
TCONS_00138408	chr7:149761434-149764025	7	-	ENSMUST00000152754.1	H19	Intergenic
TCONS_00135722	chr7:80688957-80703006	5	-	ENSMUST00000184554.7	1810026B05Rik-1_1	Intergenic
TCONS_00033321	chr12:52234327-52235648	2	-	-	lnc-SNORA25-1	Intergenic
TCONS_00157158	chrX:150158133-150175807	4	+	ENSMUST00000140575.1	2210013O21Rik	Intergenic
TCONS_00116368	chr5:123583732-123588118	3	-	ENSMUST00000159072.1	AI480526	Intergenic
TCONS_00148153	chr9:72887535-72890554	2	+	-	lnc-Pigb-1	divergent
TCONS_00152397	chr9:88415894-88417721	5	-	ENSMUST00000034997.6	Snhg5	Intergenic
TCONS_00022190	chr11:116938104-116940269	2	+	ENSMUST00000149822.1	2810008D09Rik	Intergenic
TCONS_00068890	chr19:8797802-8800934	10	+	ENSMUST00000166221.1	Snhg1	divergent
TCONS_00062922	chr17:35087188-35089413	5	-	ENSMUST00000172501.1	1110038B12Rik	Intergenic
TCONS_00115907	chr5:115552931-115557902	2	-	ENSMUST00000100847.3	Gm13826	Intergenic
TCONS_00030957	chr12:70223960-70224805	2	+	-	lnc-Gm15571-1	Intergenic
TCONS_00089833	chr3:88032230-88033882	3	+	-	lnc-Gm3764-1_1	Intergenic
TCONS_00031293	chr12:80909078-80915334	2	+	-	lnc-Rad51l1-1	Intergenic
TCONS_00115158	chr5:100849861-100858532	5	-	ENSMUST00000145557.1	5430416N02Rik	Intergenic
TCONS_00036801	chr13:83867069-83868718	1	+	ENSMUST00000171323.1	C130071C03Rik	Intergenic
TCONS_00140370	chr8:73300708-73301478	1	+	ENSMUST00000191396.1	2010320M18Rik	convergent
TCONS_00092515	chr3:26052404-26052992	1	-	-	lnc-Nlgn1-1_2	Intergenic
TCONS_00076980	chr2:118571494-118574546	1	+	ENSMUST00000149978.1	5430417L22Rik	Intergenic
TCONS_00039739	chr13:84164827-84206180	2	-	-	Gm17750	Intergenic
TCONS_00065369	chr18:33954575-33955640	3	+	ENSMUST00000146010.1	2410004N09Rik	convergent
TCONS_00008194	chr1:169615816-169620086	2	-	ENSMUST00000111377.1	lnc-Lmx1a-1	overlapping antisense
TCONS_00119529	chr6:65100926-65101303	1	+	-	lnc-Hpgds-1	Intergenic
TCONS_00109602	chr5:74489102-74490362	3	+	ENSMUST00000120364.1	2700023E23Rik	Intergenic
TCONS_00104851	chr4:109649352-109650096	2	-	ENSMUST00000124754.1	3010003L10Rik	divergent
TCONS_00035981	chr13:49063656-49065243	2	+	ENSMUST00000146582.1	C030044B11Rik	divergent
TCONS_00085622	chr2:127617124-127618224	2	-	ENSMUST00000135091.1	1500011K16Rik	Intergenic
TCONS_00015326	chr10:91618180-91628236	4	-	ENSMUST00000137229.1	Rmst	Intergenic
TCONS_00076350	chr2:98506700-98507489	1	+	ENSMUST00000099683.1	lnc-Gm10800-1	overlapping antisense
TCONS_00015654	chr10:109651771-110020376	3	-	-	lnc-E2f7-1	Intergenic
TCONS_00142702	chr8:19980818-19981101	1	-	ENSMUST00000168202.1	2610005L07Rik-1	Intergenic
TCONS_00032117	chr12:110871445-110882817	4	+	ENSMUST00000165010.1	Rian	Intergenic
TCONS_00032188	chr12:110973191-110987665	8	+	ENSMUST00000167157.1	Mirg	Intergenic
TCONS_00042643	chr14:65206952-65212830	4	+	ENSMUST00000164645.1	A930011O12Rik	Intergenic
TCONS_00054223	chr16:53985554-53986226	1	+	-	lnc-AC125028.1	Intergenic
TCONS_00138641	chr8:3622428-3625471	4	+	ENSMUST00000156380.1	2900053A13Rik	overlapping antisense
TCONS_00129394	chr7:86660118-86677474	2	+	ENSMUST00000150985.1	AI854517	Intergenic
TCONS_00019565	chr11:77303356-77304911	2	+	ENSMUST00000171677.1	2210008F06Rik	Intergenic
TCONS_00002664	chr1:122518213-122586980	8	+	-	lnc-En1-1_3	Intergenic
TCONS_00148989	chr9:106051018-106054081	2	+	ENSMUST00000143956.1	D030055H07Rik	Intergenic
TCONS_00119247	chr6:52052948-52062531	3	+	ENSMUST00000125374.1	Gm15055	Intergenic
TCONS_00072147	chr19:54004167-54018661	4	-	ENSMUST00000148690.1	Bbip1	overlapping antisense
TCONS_00126797	chr7:16633660-16634413	1	+	-	lnc-Zfp541-1_1	Intergenic
TCONS_00049031	chr15:103076014-103077127	2	+	ENSMUST00000087351	Hnrnpa1	Intergenic
TCONS_00120094	chr6:86477395-86514436	5	+	ENSMUST00000128779.1	1600020E01Rik	Intergenic
TCONS_00086213	chr2:147762511-147865939	9	-	ENSMUST00000129082.1	9030622O22Rik	Intergenic
TCONS_00004620	chr1:4677948-4679320	1	-	-	lnc-U6-1_4	Intergenic
TCONS_00135414	chr7:69058470-69077736	5	-	ENSMUST00000137237.1	A330076H08Rik	Intergenic

TCONS_00124588	chr6:113020392-113027204	2	-	ENSMUST00000124246.3	Gt(ROSA)26Sor	divergent
TCONS_00013480	chr10:36859252-36861073	2	-	ENSMUST00000062667.3	lnc-5930403N24Rik-1	overlapping antisense
TCONS_00032463	chr12:120591340-120591768	1	+	-	lnc-Gm6768-1	Intergenic
TCONS_00055859	chr16:21331970-21333413	1	-	76222.1 (refSeq)	Magef1	Intergenic
TCONS_00080252	chr2:180454525-180456872	2	+	-	lnc-2310003C23Rik-1	Intergenic
TCONS_00046552	chr15:8395177-8395858	1	+	-	lnc-Nipbl-1_2	divergent
TCONS_00158266	chrX:35533292-35533923	1	-	ENSMUST00000118349.1	lnc-Gm14556-1	Intergenic
TCONS_00063741	chr17:46993166-46994043	1	-	-	lnc-BC032203-1	Intergenic
TCONS_00156731	chrX:136965424-136970007	2	+	-	lnc-Gm8097-1	Intergenic
TCONS_00004331	chr1:183950276-183959960	2	+	-	lnc-Enah-1_2	divergent
TCONS_00075420	chr2:72817481-72826734	3	+	ENSMUST00000123087.1	1700011J10Rik	overlapping antisense
TCONS_00130838	chr7:123587063-123587841	1	+	-	lnc-Pik3c2a-1	Intergenic
TCONS_00126450	chr7:6706061-6707220	1	+	-	lnc-Peg3-1	Intergenic
TCONS_00160880	chrX:148729370-148762038	3	-	ENSMUST00000148326.1	2900056M20Rik	Intergenic
TCONS_00052924	chr16:21794435-21809110	3	+	ENSMUST00000151043.1	1300002E11Rik	Intergenic
TCONS_00069468	chr19:33466203-33467304	1	+	ENSMUST00000163093.1	lnc-Rnls-1	overlapping antisense
TCONS_00152101	chr9:72827531-72833183	2	-	ENSMUST00000124565.1	Gm5918	divergent
TCONS_00154256	chrX:20937991-20941748	2	+	ENSMUST00000127554.1	Gm5124	Intergenic
TCONS_00123013	chr6:47381784-47383146	2	-	-	lnc-Cul1-1	Intergenic
TCONS_00083290	chr2:71368650-71375948	2	-	ENSMUST00000137251.2	Dlx1as	overlapping antisense
TCONS_00001682	chr1:75195223-75195974	3	+	ENSMUST00000145459.1	Ankzf1	overlapping antisense
TCONS_00129509	chr7:88668541-88676385	4	+	ENSMUST00000143530.1	2900076A07Rik	Intergenic
TCONS_00038691	chr13:49436219-49436720	1	-	-	lnc-Bicd2-1	divergent
TCONS_00152261	chr9:79914299-79914634	1	-	-	lnc-Senp6-1_2	divergent
TCONS_00048473	chr15:89875438-89905660	6	+	ENSMUST00000082986.1	lnc-U6-1_11	Intergenic
TCONS_00117334	chr5:143200556-143206004	3	-	-	lnc-Slc29a4-1	Intergenic
TCONS_00034375	chr12:100801028-100801739	1	-	-	lnc-Gm16956-1	Intergenic
TCONS_00159850	chrX:100655711-100678915	8	-	ENSMUST00000127786.1	Xist	Intergenic
TCONS_00092846	chr3:40689819-40698045	3	-	ENSMUST00000149173.1	6430590A07Rik	overlapping antisense
TCONS_00140569	chr8:75016833-75055466	2	+	ENSMUST00000167312.1	Gm17435	overlapping antisense
TCONS_00033795	chr12:79326111-79327505	1	-	-	lnc-Gphn-1	divergent
TCONS_00065449	chr18:35713064-35717970	4	+	ENSMUST00000166243.1	Snhg4	Intergenic
TCONS_00068467	chr19:4125989-4131727	6	+	ENSMUST00000140405.2	Tmem134	divergent
TCONS_00000415	chr1:30827140-30828646	5	+	-	lnc-Phf3-1	Intergenic
TCONS_00144005	chr8:55605787-55615351	5	-	ENSMUST00000152597.1	Spcs3	Intergenic
TCONS_00042776	chr14:68743028-68743543	1	+	-	A230070E04Rik	divergent
TCONS_00151396	chr9:54672021-54673162	2	-	-	lnc-Ireb2-1	Intergenic
TCONS_00070743	chr19:6530711-6532011	2	-	ENSMUST00000149574.1	Gm14964	overlapping antisense
TCONS_00056047	chr16:28752921-28753322	2	-	-	lnc-1600021P15Rik-1_2	Intergenic
TCONS_00051980	chr16:6349112-6488226	2	+	-	lnc-Rbfox1-1_1	Intergenic
TCONS_00089311	chr3:63709353-63709731	1	+	-	lnc-snoU13-1	Intergenic

Supplementary Table S2

Most significant SNP	Nearest gene	lncRNA from DA repertoire
rs6430538	TMEM163,CCNT2	2900009J06Rik-1
rs11060180	OGFOD2	Gm16001-1
rs115185635	CHMP2B	**lnc-Chmp2b-1**
rs4784227	TOX3	**lnc-Tox3-1_2**
rs14235	ZNF646	**lnc-Fbxl19-1**
rs1555399	TMEM229B	**lnc-Arg2-1**
rs329648	MIR4697	**lnc-Gm17254-1**
rs356182	SNCA	**lnc-Snca-1**

Supplementary Table S3

gene name	forward 5'-3'	reverse 5'-3'
Lmx1a	ctgcagaagggtgacgagtt	gtggtcaggatggttctggg
Lmx1b	aaaggcagtggagatgacgg	tctgctgccaatgtctctcg
En1	gggtctactgcacacgctatt	ttttcttctttagcttcctggtg
En2	gctattctgaccggccttct	gactcgttcaggctgagctc
Nr4a2	gcctagctgttgggatggtt	cgagggcactgatcagactc
Foxa2	gtgaagatggaagggcacga	tcattccagcgcccacatag
Th	gcctcctcacctatgcactc	ctgggagaactgggcaaatg
Dat	ctccaccctcatcaacccac	atcttccagacaccagcagc
Vmat2	gctcctcaccaacccattca	cagccacagatgagcaagag
Kcnj6	gttccgagagaggcgatcag	atcctggtccatggaatcgc
Tph2	ctgaatccgcctgagagcat	ccgtacatgaggactcggtg
Gfap	aaagactgtggagatgcggg	aaggaagggaagtgctggtg
1810026B05Rik-1	agatggaatgaaaggcctgca	tgggctgccatcttgatttct
lnc-Enah-1	cctgcctcactcttccttgg	tcaagctgtgtacacgaccc
Rmst	tttgagagctcggcccattt	ctcaggatcgcggtgacaat
2010320M18Rik	aaccgaacgacctcaactcc	tcgccttccattccttccac
lnc-Plxna2	gtgcaagctatacccctgca	agagggcagtaggggacatt
Gm2694	cagcctaacgtctccaagca	tacgtgctttggacctctgc
Kantr	tgcatcctctccagacggta	tcggaggagtctgtgttcca
lnc-5930403N24Rik-1	ttctcgcctccatcgttcac	ttgccaccgaagtaccagac
2900009J06Rik	tcttgtcattgggagacgcc	gctggactagggttgggaac
lnc-Nkain2-1	ccactggaaaacctgcatgc	ttccgttcggtccttgtagc
lnc-Hpgds-1	acacaaatgccagaccccaa	acagtagagccaaaggtgcc
lnc-Pik3c2a	ctttcattccctccagcggt	ctgtcagcaaacaccaaggc
1700045I19Rik	tgagcccaccttcgattgtc	gccccatggaagagataggc
C130071C03Rik	cgcatagccatctgaccact	tggcattttccagccttacct
lnc-En1	gttcgaagtctaccagggct	ttcacacaaaagcccactgg
1810044D09Rik	tggaaaaggaacactgccca	gcgtgtgaaggagaaacagc
lnc-U6_11	gagaacaactgccccaaaagg	aatgcaggtggagtcaggc
lnc-Gm6768	cccatacccaagaccaaccc	ctagtttggggctgcagtga
Snhg4	cagtttgaggcgatgtctgc	ctagaaccacagtgcaccct
lnc-Slc25a24-1	aattccccacggttgatgct	tttggggatgggatgggttg
lnc-U6-1_4	gagccaagttcaagcgcaaa	aactttattgaggggcgggg

A930011O12Rik	tacacccggaccctacactc	gaccaccgcatccttcctac
lnc-Slc25a24-1	ctggagctggtgttcgagtt	tcaagaagcctcgaccacac
lnc-BC032203-1	gtttgctttcggtcgcttgt	tccttagcgttgtcagctgg
Snhg1	aggatgggtgtacgctctct	ctggtacggctcctttgttc
lnc-Cpsf2-1	ccagtggcctagctccct	catccaaagacaaatcccgca
Snhg5	cgttcacaaggacaatggcg	ggcatctcactggtcagcat
2700069I18Rik-1	agataatgtccgagccgctg	ctttcggccgagcagttttc

Supplementary Table S4

a

	U	p
Lmx1a	3	0.2
Lmx1b	0	0.0286
En1	0	0.0286
En2	7	0.8286
Nr4a2	0	0.0286
Foxa2	6	0.6571
Th	7	0.8286
Dat	0	0.0286
Vmat2	3	0.2
Kcnj6	0	0.0286
Tph2	0	0.0286
Gfap	0	0.0286

b

	U	p
1810026B05Rik-1_1	5	0.4857
lnc-Enah-1_2	5	0.4857
Rmst	1	0.0571
2010320M18Rik	5.5	0.5143
lnc-Plxna2-1	0	0.0286
Gm2694	3.5	0.2286
Kantr	7	0.8286
lnc-5930403N24Rik-1	0	0.0286
2900009J06Rik	0	0.0286
lnc-Nkain2-1	0	0.0286
lnc-Hpgds-1	1	0.0571
lnc-Pik3c2a-1	6	0.6571
1700045I19Rik	2	0.1143
C130071C03Rik-1	2	0.1143
lnc-En1-1_3	0	0.0286
1810044D09Rik	0	0.0286
lnc-U6-1_11	0	0.0286
lnc-Gm6768-1	0	0.0286
Snhg4	0	0.0286
lnc-Slc25a24-1	0	0.0286
lnc-U6-1_4	6	0.6571
A930011O12Rik	2	0.1143
lnc-Zfp541-1_1	3	0.2
lnc-BC032203-1	7	0.8286
Snhg1	0	0.0286
lnc-Cpsf2-1	0	0.0286
Snhg5	4	0.3429
2700069I18Rik-1_2	2	0.1143

c

	U	p
Snhg5	8	0.3413
lnc-En1-1_3	0	0.0079
lnc-U6-1_11	0	0.0079
lnc-Hpgds-1	8	0.3413
2900009J06Rik	2	0.0317
Snhg1	6	0.1984

SUPPLEMENTARY FIGURE LEGENDS

Supplementary Fig. S1. Normalized read coverage along the 5'-3' sequences of all known Refseq expressed transcripts in our datasets.
The sequence position is normalized from 0 to 100%. 0 corresponds to the 5'end and 100 to the 3'end. Independent plots corresponding to the 3 DA RNA-seq samples were generated according to the transcript length. These data clearly confirm the existence of a technology-associated bias that implies that transcription start sites (TSS) are underrepresented in our sequencing datasets, especially for long transcripts, independent of the number of exons.

Supplementary Fig. S2. FACS sorting from Masch1 CRE X Rosa YFP mice E14.5 embryos allows for efficient enrichment in 5-HT neurons. a- FACS sorting of YFP^+ and YFP^- cell populations for subsequent RNA-seq and ATAC-seq analyses. **b-** Tph2 expression (red) assessed by immunofluorescence on YFP^+ and YFP^- cells cultured for 90 minutes after FACS. Nuclei were stained using Hoechst (blue). **c-** *Tph2* mRNA relative expression of 3 independent YFP^+ cell populations (triangle, diamond and square) used for RNA-seq, compared to their matching control YFP^- cell populations. The bar represents the mean of the 3 enrichment values. mRNA expression was normalized relatively to *Tbp* mRNA expression. **d-** mRNA expression in Log10(FPKM) of numerous cellular subtype marker genes. Each circle represents mRNA expression of a marker gene from 1 RNA-seq, and the bar represents the mean of the 3 values. Error bars show standard error of the mean. The color code is as follow: green, serotonergic neurons; yellow, dopaminergic neurons; blue, GABAergic neurons; purple, glutamatergic neurons; mustard, noradrenergic neurons; brown, radial glial like cells.

Supplementary Fig. S3. Principal component analysis (PCA). PCA1, represented by the X-axis, segregates DA neurons (blue) from the 5-HT neurons (orange), with 86% of the variance between all the datasets. PCA2, represented by the Y-axis, illustrates 7% of the variance between the samples. Of note, one replicate from the 5-HT datasets segregated from the 2 others in PCA2, and corresponds to a dataset in which less reads were mapped.

Supplementary Fig. S4. Identification of the lncRNAs expressed in the 5-HT neurons from hindbrain rhombomeres r1-r3 of E14.5 embryos. Number of lncRNAs depending on their categories. Dark grey, lncRNAs annotated in Ensembl; light grey, lncRNAs unannotated.

Supplementary Fig. S5. Schematics of the locus of selected lncRNA candidates and their comparative expression in E14.5 embryos ventral mesencephalons and rhombomeres1-3. a- *Snhg5* (intergenic, annotated); **a'-** enlargement of **a**; **a''-** *Snhg5* expression; **b-** *lnc-EN1-1_3* (intergenic, unannotated); **b'-** enlargement of **b**; **b''-** *lnc-EN1-1_3* expression; **c-** *lnc-U6-1_11* (intergenic, unannotated); **c'-** enlargement of **c**; **c''-** *lnc-U6-1_11* expression; **d-** *lnc-Hpgds-1* (intergenic, unannotated); **d'-** *lnc-Hpgds-1* expression; **e-** *2900009J06Rik* (overlapping antisense, annotated); **e'-** *2900009J06Rik* expression; **f-** *Snhg1* (divergent, annotated); **f'-** *Snhg1* expression. LncRNAs are represented in black if not annotated or in dark blue if annotated. ATAC-seq peaks are depicted in black, reads from RNA-seq are in light blue for the DA minus strand, dark blue for the DA plus strand and orange for the 5-HT track (unstranded). The scales represent reads per millions (RPM). a'', b", c", d', e' and f'- Expression experiments have been performed on 5 independent samples containing each 5 ventral mesencephalons (DA) and 4 independent samples containing each 5 rhombomeres r1-3 (5-HT) of E14.5 embryos. RNA expression from each sample is depicted by black circles and grey columns represent the mean values. RNA expression was normalized relatively to *Tbp* mRNA expression and to the mean of the DA samples. Error bars show standard error of the mean. * p-value ≤ 0.05 ; ** p-value ≤ 0.01.

Supplementary Figure S1

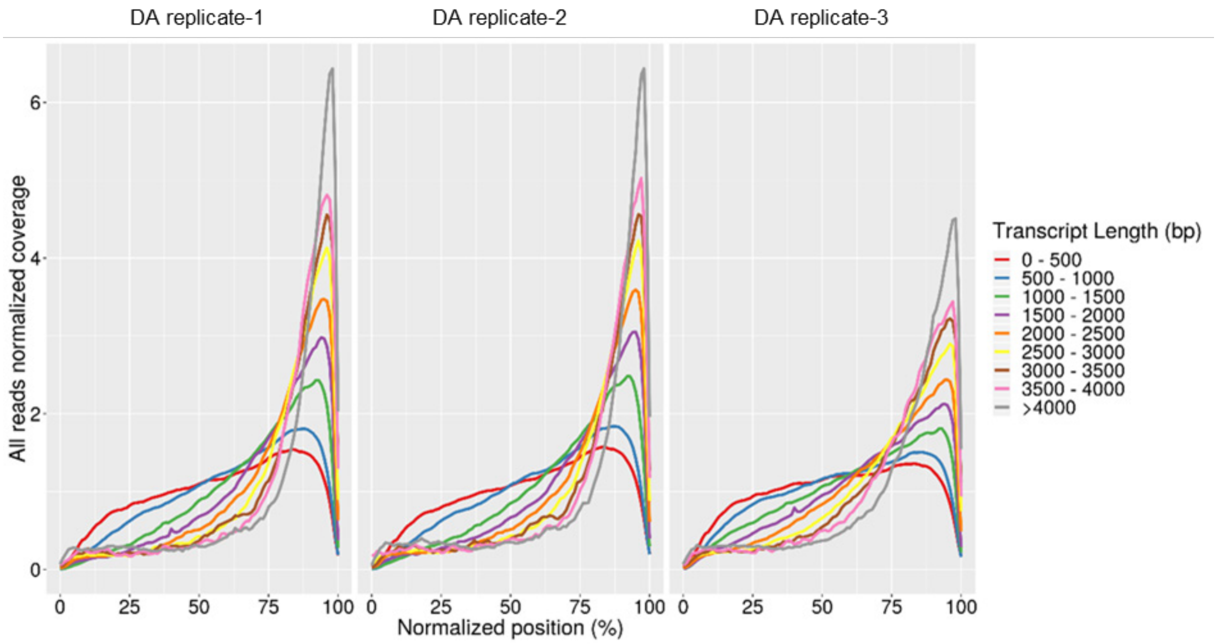

Supplementary Figure S2

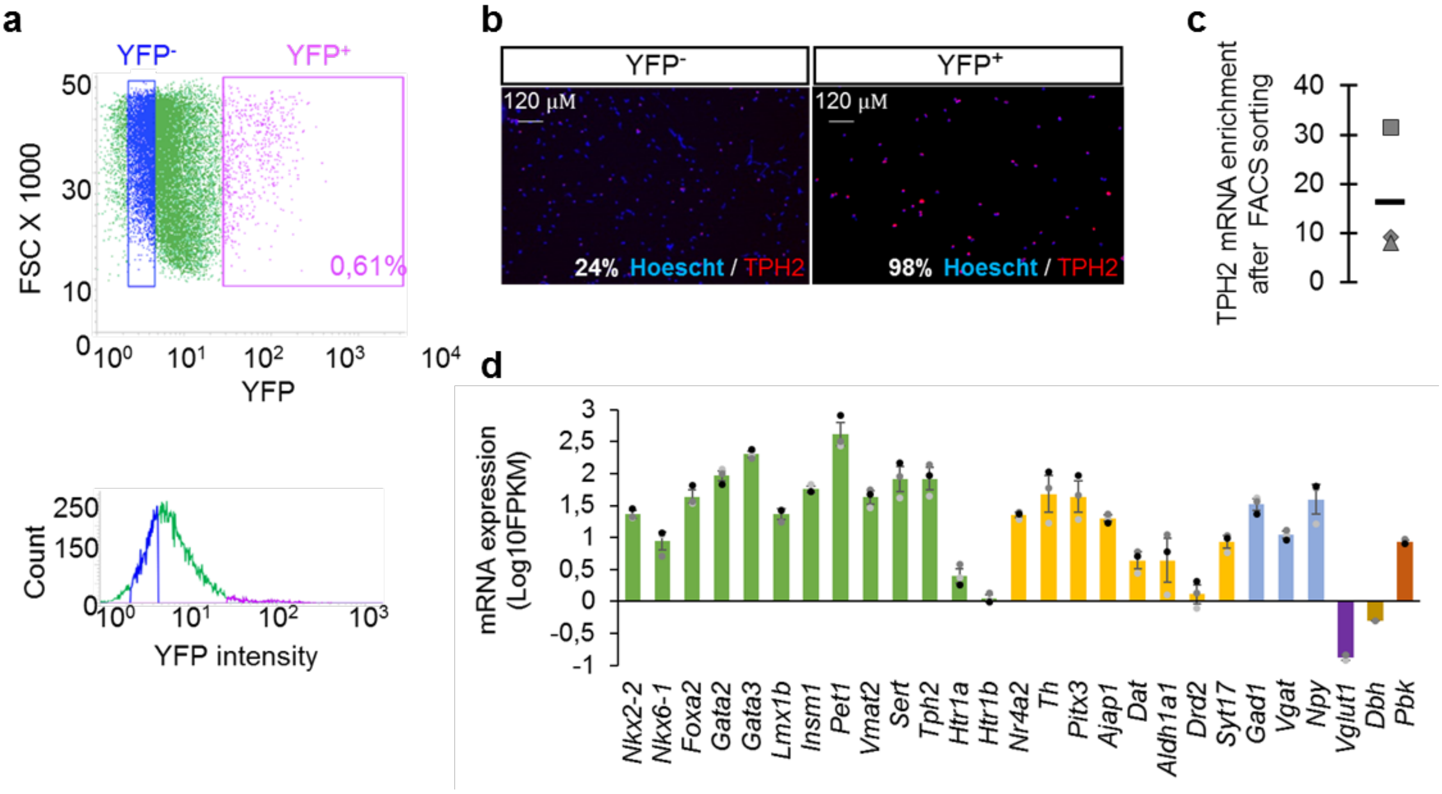

Supplementary Figure S3

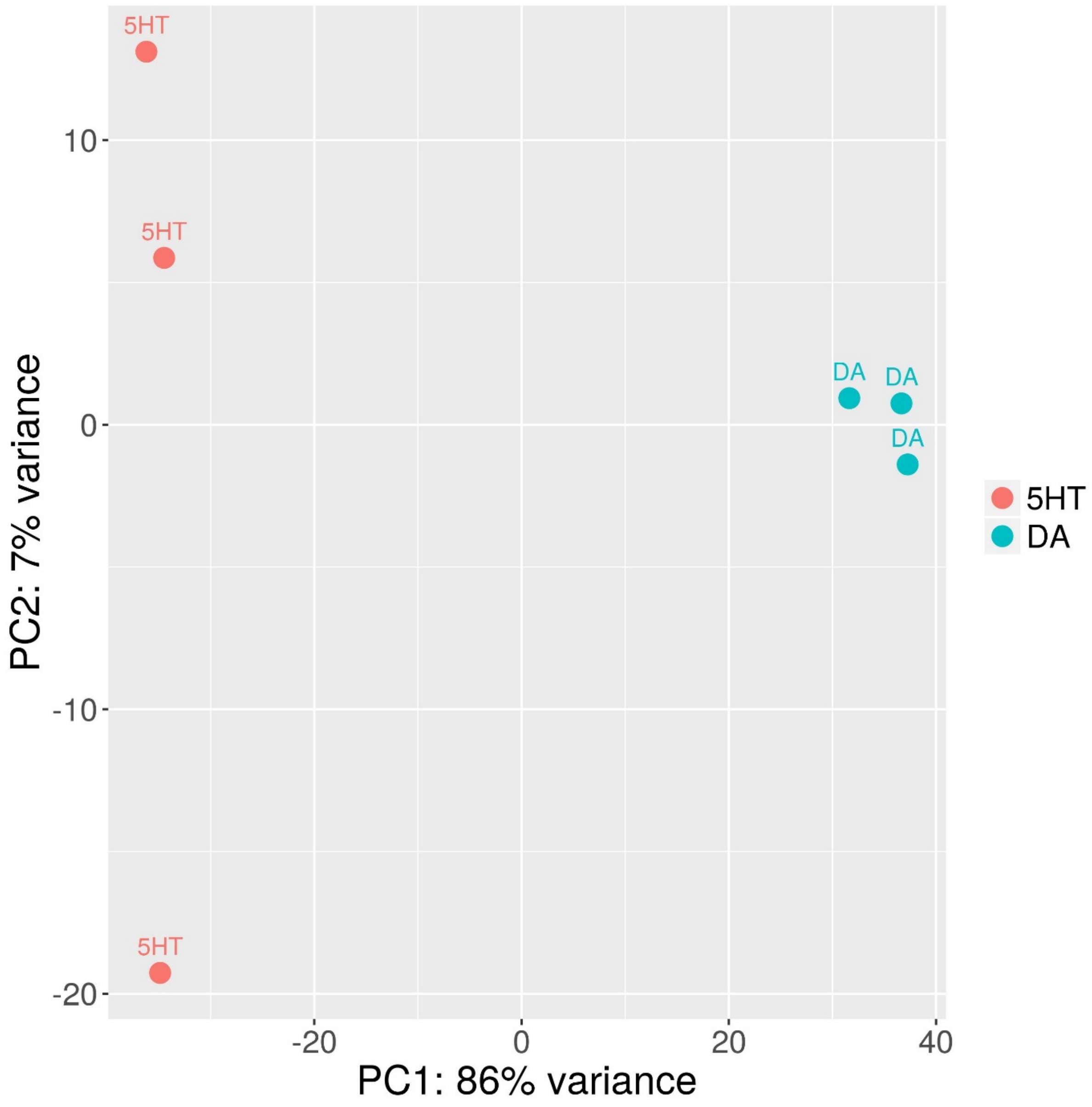

Supplementary Figure S4

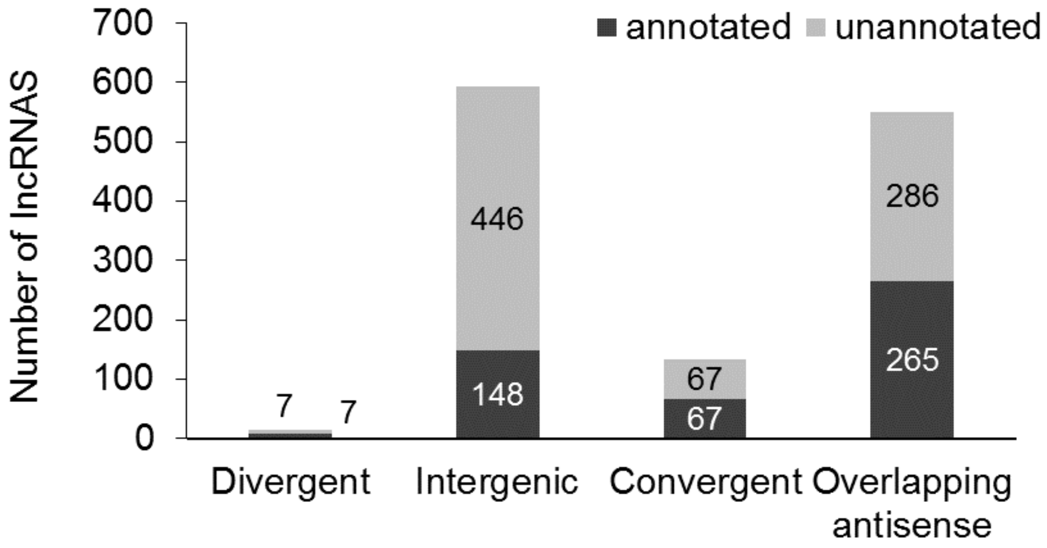

Supplementary Figure S5

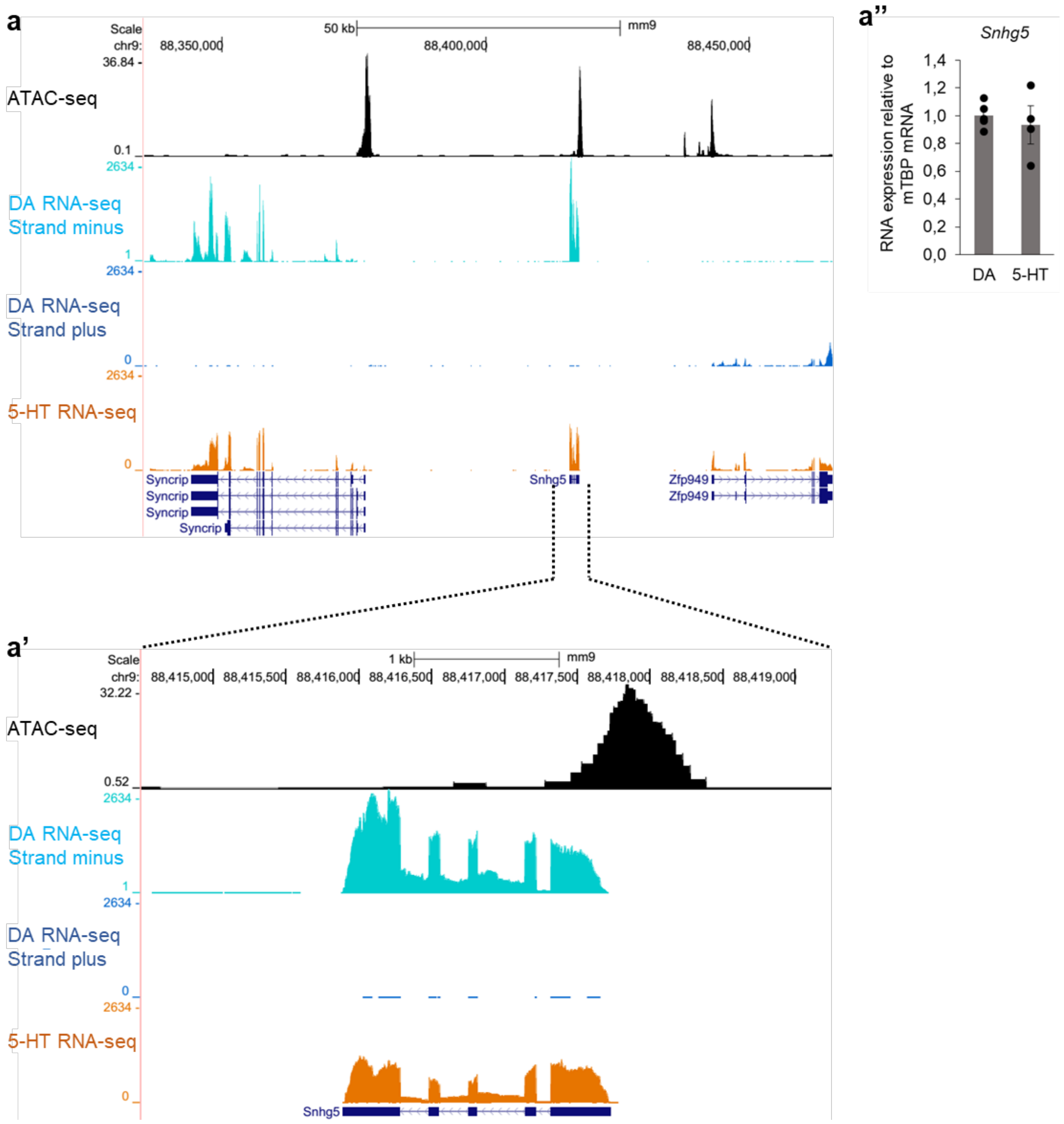

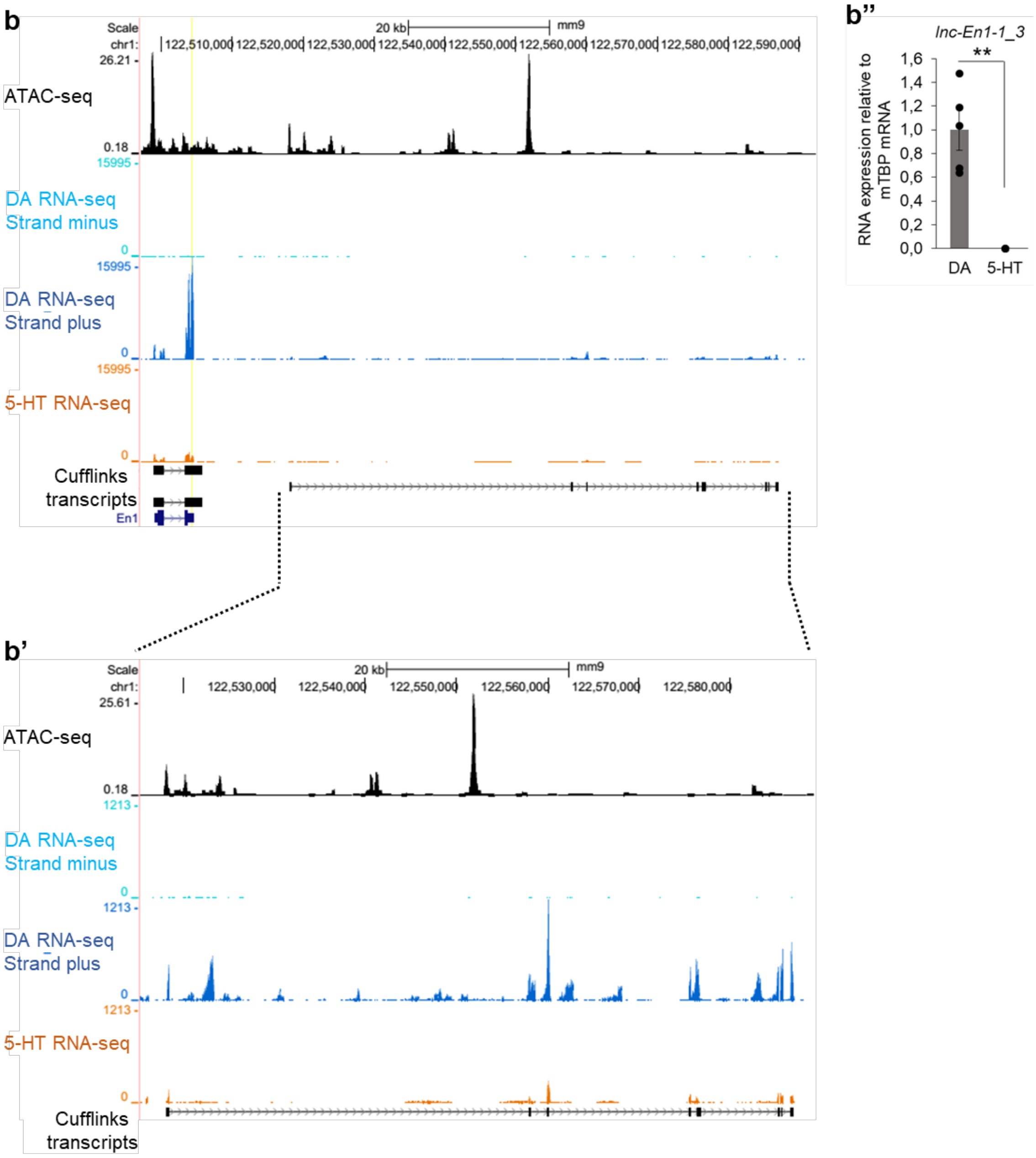
b
Scale
chr1:
20 kb
mm9
122,510,000
122,520,000
122,530,000
122,540,000
122,550,000
122,560,000
122,570,000
122,580,000
122,590,000
26.21
ATAC-seq
0.18
15995
DA RNA-seq
Strand minus
0
15995
DA RNA-seq
Strand plus
0
15995
5-HT RNA-seq
0
Cufflinks
transcripts
En1
b'
Scale
chr1:
20 kb
mm9
122,530,000
122,540,000
122,550,000
122,560,000
122,570,000
122,580,000
25.61
ATAC-seq
0.18
1213
DA RNA-seq
Strand minus
0
1213
DA RNA-seq
Strand plus
0
1213
5-HT RNA-seq
0
Cufflinks
transcripts
b''
lnc-En1-1_3
RNA expression relative to
mTBP mRNA
1,6
1,4
1,2
1,0
0,8
0,6
0,4
0,2
0,0
**
DA
5-HT

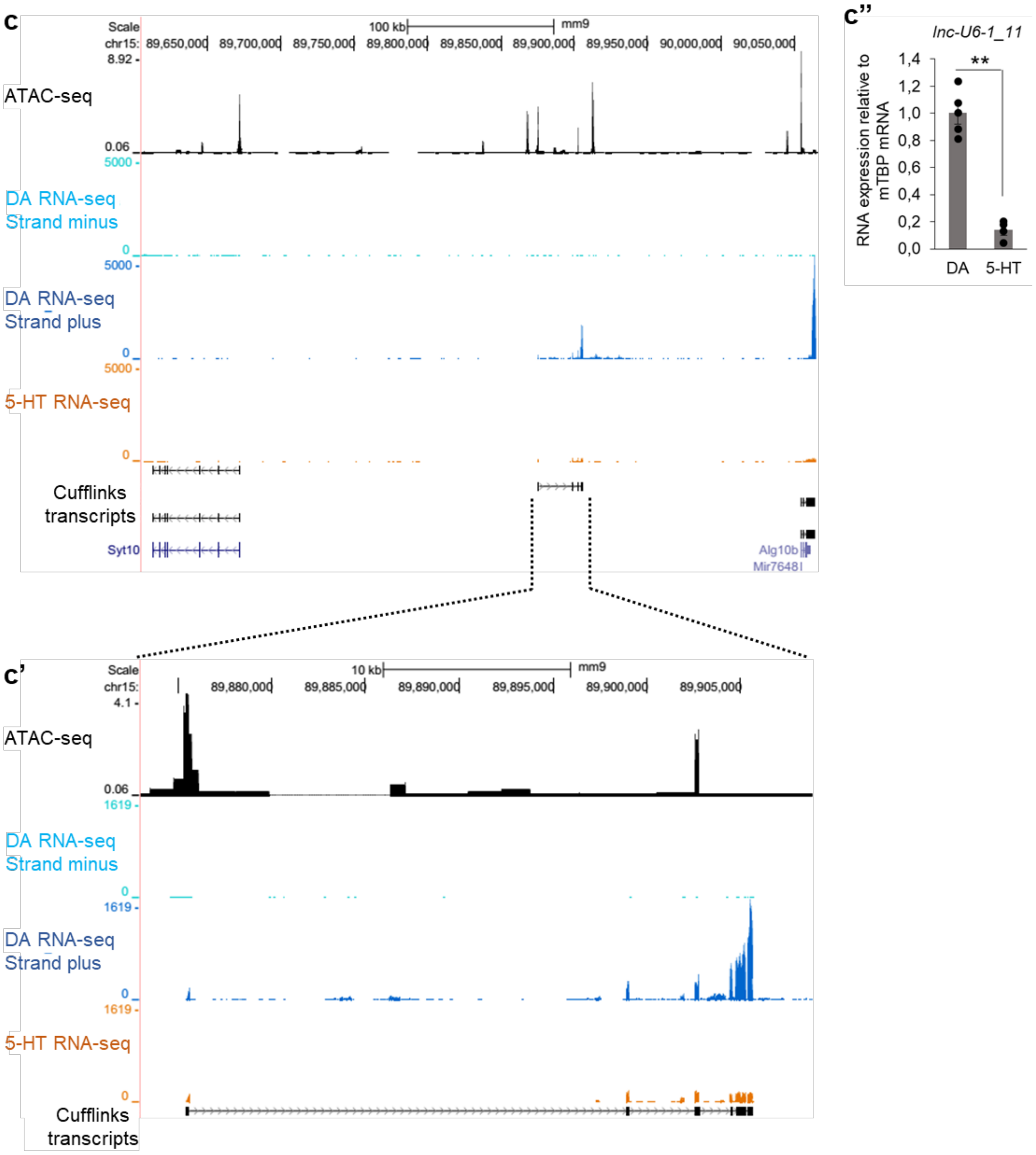
c
Scale
chr15:
100 kb
mm9
89,650,000
89,700,000
89,750,000
89,800,000
89,850,000
89,900,000
89,950,000
90,000,000
90,050,000
ATAC-seq
DA RNA-seq Strand minus
DA RNA-seq Strand plus
5-HT RNA-seq
Cufflinks transcripts
Syt10
Alg10b
Mir7648
c'
10 kb
89,880,000
89,885,000
89,890,000
89,895,000
89,900,000
89,905,000
c''
lnc-U6-1_11
RNA expression relative to mTBP mRNA
**
DA
5-HT

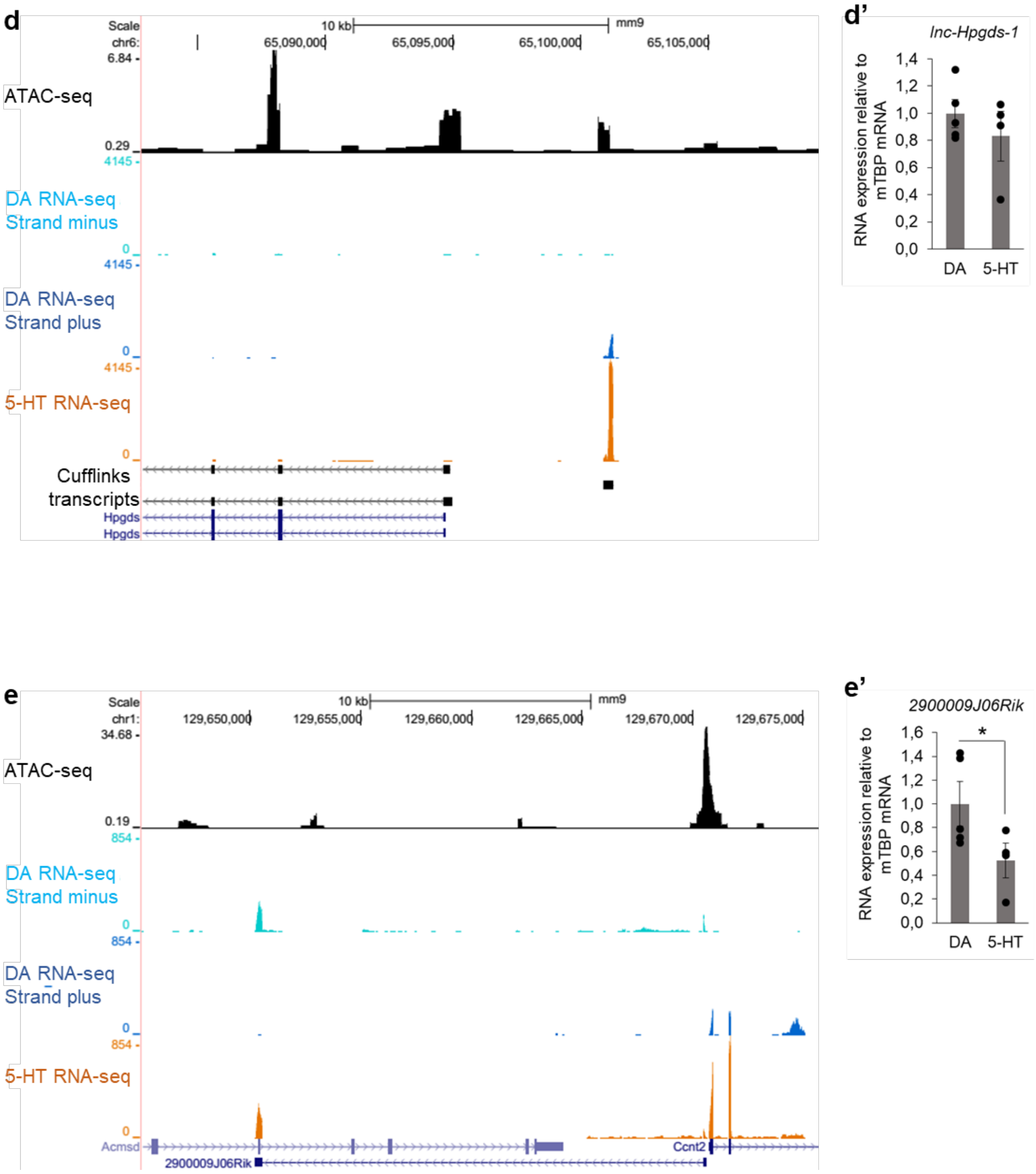
d
Scale
chr6:
10 kb
mm9
65,090,000
65,095,000
65,100,000
65,105,000
6.84
ATAC-seq
0.29
4145
DA RNA-seq
Strand minus
0
4145
DA RNA-seq
Strand plus
0
4145
5-HT RNA-seq
0
Cufflinks
transcripts
Hpgds
Hpgds
d'
lnc-Hpgds-1
RNA expression relative to
mTBP mRNA
1,4
1,2
1,0
0,8
0,6
0,4
0,2
0,0
DA
5-HT
e
Scale
chr1:
10 kb
mm9
129,650,000
129,655,000
129,660,000
129,665,000
129,670,000
129,675,000
34.68
ATAC-seq
0.19
854
DA RNA-seq
Strand minus
0
854
DA RNA-seq
Strand plus
0
854
5-HT RNA-seq
0
Acmsd
Ccnt2
2900009J06Rik
e'
2900009J06Rik
RNA expression relative to
mTBP mRNA
1,6
1,4
1,2
1,0
0,8
0,6
0,4
0,2
0,0
*
DA
5-HT

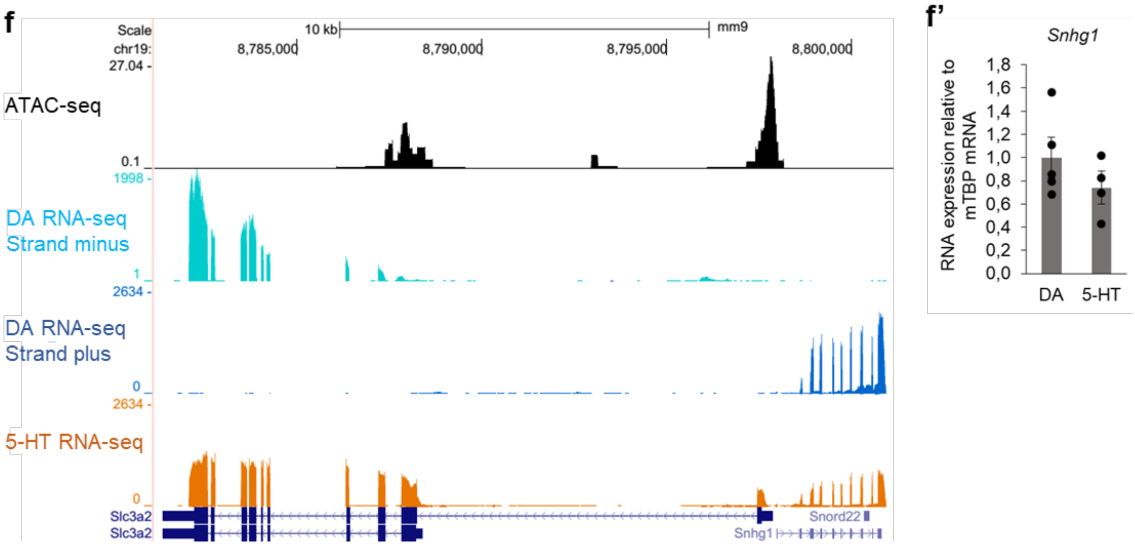
f
Scale
chr19:
10 kb
mm9
8,785,000
8,790,000
8,795,000
8,800,000
27.04 -
ATAC-seq
0.1
1998 -
DA RNA-seq
Strand minus
1
2634 -
DA RNA-seq
Strand plus
0
2634 -
5-HT RNA-seq
0
Slc3a2
Slc3a2
Snord22
Snhg1
f'
Snhg1
RNA expression relative to
mTBP mRNA
1,8
1,6
1,4
1,2
1,0
0,8
0,6
0,4
0,2
0,0
DA
5-HT
